# Transcription activity contributes to the firing of non-constitutive origins in African trypanosomes helping to maintain robustness in S-phase duration

**DOI:** 10.1038/s41598-019-54366-w

**Published:** 2019-12-06

**Authors:** Marcelo S. da Silva, Gustavo R. Cayres-Silva, Marcela O. Vitarelli, Paula A. Marin, Priscila M. Hiraiwa, Christiane B. Araújo, Bruno B. Scholl, Andrea R. Ávila, Richard McCulloch, Marcelo S. Reis, Maria Carolina Elias

**Affiliations:** 1 Laboratório Especial de Ciclo Celular, Center of Toxins, Immune Response and Cell Signaling (CeTICS), Instituto Butantan, São Paulo, Brazil; 2 Plataforma de citometria de fluxo, Instituto Carlos Chagas, FIOCRUZ, Paraná, Brazil; 3 Laboratório de Regulação da Expressão Gênica, Instituto Carlos Chagas, FIOCRUZ, Paraná, Brazil; 4 The Wellcome Centre for Molecular Parasitology, Institute of Infection, Immunity and Inflammation, University of Glasgow, Glasgow, United Kingdom

**Keywords:** Parasite biology, Parasite genetics, Double-strand DNA breaks, DNA synthesis, Origin firing

## Abstract

The co-synthesis of DNA and RNA potentially generates conflicts between replication and transcription, which can lead to genomic instability. In trypanosomatids, eukaryotic parasites that perform polycistronic transcription, this phenomenon and its consequences are still little studied. Here, we showed that the number of constitutive origins mapped in the *Trypanosoma brucei* genome is less than the minimum required to complete replication within S-phase duration. By the development of a mechanistic model of DNA replication considering replication-transcription conflicts and using immunofluorescence assays and DNA combing approaches, we demonstrated that the activation of non-constitutive (backup) origins are indispensable for replication to be completed within S-phase period. Together, our findings suggest that transcription activity during S phase generates R-loops, which contributes to the emergence of DNA lesions, leading to the firing of backup origins that help maintain robustness in S-phase duration. The usage of this increased pool of origins, contributing to the maintenance of DNA replication, seems to be of paramount importance for the survival of this parasite that affects million people around the world.

## Introduction

DNA replication is an essential tightly regulated process, basis of genetic inheritance, which follows a specific temporal program. In cellular organisms, the earliest step in DNA replication is the establishment of origins that are defined as genomic loci, where DNA synthesis initiates. In eukaryotes, replication initiation is preceded by the binding of an initiator called the Origin Recognition Complex (ORC)^1^. This complex establishes replication initiation through the recruitment and activation of the replisome; hereafter, we refer to this phenomenon as origin firing. An origin firing yields two replication forks, which proceed in opposite directions (bi-directional movement) following a DNA replication rate that varies according to the cell type^1–3^. The time required to complete DNA replication on all chromosomes determines the S-phase duration, which can be one of the primary methods to regulate cell cycle progression in eukaryotes^4,5^. Regarding the number of origins per chromosome, bacteria typically have a single origin, whereas eukaryotes and Archaea generally have multiple origins per chromosome^1,6^, although the exact number varies according to cell type and the cellular environment.

During the G1 phase of the cell cycle, all potential replication origins are licensed, *i.e*., the loading of DNA helicase core (namely, MCM_2–7_) is carried out^6,7^. However, during the progression of a single cell cycle, only a subset of these origins activates DNA synthesis. The choice of origins that will be activated in the cell cycle varies from cell to cell, which implies flexibility regarding origin usage^6^. According to their different usages, replication origins can be classified into three categories: constitutive, which are always activated in all cells of a given population; flexible, whose usage varies from cell to cell; or dormant, which are not fired during a normal cell cycle but are activated in the presence of replication stress^6^. A remarkable phenomenon that may contribute to replication stress is replication-transcription conflicts. These clashes are a powerful source of genomic instability that can generate DNA double-stranded breaks (DSBs), which in turn impair DNA synthesis^8–10^. Whether replication-transcription conflicts also contribute to the activation of origins to avoid lengthening S phase and consequent impairment of cell cycle time is a question that remains open.

In trypanosomatids, unlike eukaryotic model organisms, the majority of genes are organized into large polycistronic clusters^11^, which makes us wonder whether these organisms regulate transcription or replication in order to mitigate potential collisions during S phase. Trypanosomatids encompass human pathogens of great medical importance, such as *Trypanosoma* spp. and *Leishmania* spp., which are the causative agents of devastating diseases that threaten millions of people around the world^12,13^. *Trypanosoma brucei*, one of the parasites known as African trypanosomes, has the best-studied DNA and RNA synthesis, although this knowledge is limited relative to that of model eukaryotes^14^. The duration of the cell cycle phases, including S phase, was recently reviewed for *T. brucei* Lister strain 427 through the usage of a most sensitive thymidine analog 5-ethynyl-2′-deoxyuridine (EdU) to monitor DNA replication^15^, though for *T. brucei* TREU927 there are still no similar assays. The number of DNA replication origins per chromosome and the replication rate are a matter of debate according to the technique used to obtain these data and the choice of either *T. brucei* Lister strain 427 or TREU927^3,14,16,17^. Even with its peculiar feature of performing polycistronic transcription in large gene clusters, thus far there have been no studies of replication-transcription conflicts in trypanosomatids.

In this work, we investigated the dynamics of origins usage in the presence of transcription activity during the S phase in *T. brucei*. We used EdU to monitor DNA replication and more accurately estimate S-phase duration. Then, based on the chromosome size, specific replication rates, and the S-phase duration, we developed a mathematical formula to estimate the minimum number of DNA replication origins required to duplicate an entire chromosome within the S-phase duration. After applying this formula to each chromosome, we compared the minimum number of origins (*mo*) obtained with the number of constitutive origins mapped by the MFA-seq technique, and we found that these values were very similar. However, when we used another formula that takes into account replication rates and the positions of mapped constitutive origins, we figured out that these mapped origins were not sufficient to allow complete replication within the S-phase duration, even in the presence of artificial origins positioned at the ends of the chromosomes. We then designed and simulated a mechanistic model of DNA replication considering origin firing in the presence or absence of polycistronic transcription during S phase. These simulations suggested robustness in S-phase duration relative to increasing levels of transcriptional activity, which were compensated by increased origin firing. Based on these results, we hypothesize that if there is transcription during S phase, then the co-synthesis of DNA and RNA can generate collisions between the two associated types of machinery, activating non-constitutive (backup) origins and consequently increasing the pool of origins used to complete S phase. Using a run-on assay, we showed the transcription landscape of the *T. brucei* cell cycle, where it was possible to observe that this organism does not limit its transcription during replication to avoid potential collisions. Moreover, we verified the presence of γH2A (a DNA lesion biomarker) and R-loops foci, partial colocalizing predominantly in late S/G2 phase. γH2A and R-loop foci decreased after transcription inhibition, and, furthermore, γH2A foci also decreased after R-loops degradation (by RNase H treatment), suggesting a role for R-loops in the formation of DNA lesions. Finally, using the DNA combing technique, we measured fewer numbers of activated origins and an increase of average replication rate after transcription inhibition. Additionally, we measured the length of S phase and observed that they remained unchanged. Together, our findings suggest that the action of the transcription machinery (probably through conflicts with replication) contributes to the activation of backup origins helping to maintain robustness in S-phase duration in *T. brucei*, a human pathogen of great medical importance.

## Results

### EdU allows a more accurate estimation of S-phase duration in *T. brucei* TREU927

To investigate the origin usage dynamics under standard situations in *T. brucei* TREU927, we first needed accurate values for S-phase duration, which could be obtained from other studies. However, our group recently published a study highlighting significant differences between the thymidine analogs BrdU and EdU, commonly used to monitor DNA replication in most organisms^15^. In summary, this study shows that EdU is much more sensitive for monitoring DNA replication than BrdU, and its usage provides a more accurate estimate of the duration of the cell cycle phases G1, S, and G2^15^. Consequently, this study pointed to skepticism regarding the accuracy of analyses performed to monitor DNA replication using BrdU (with a DNA denaturation step carried out with 2 M HCl) in trypanosomatids. Therefore, to ensure better accuracy of S-phase duration in *T. brucei* TREU927, these analyses had to be redone using EdU^18^.

**Figure 1:**
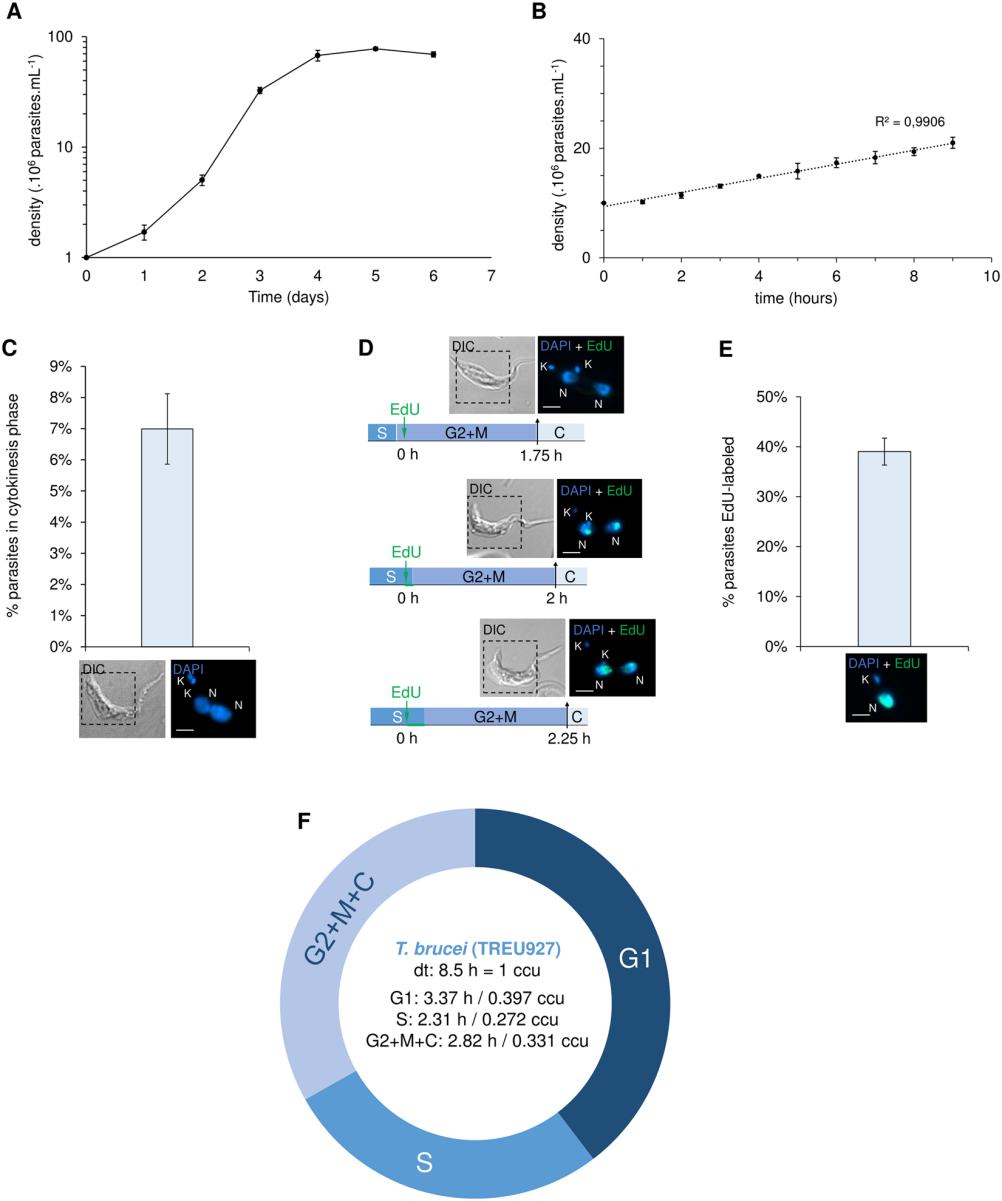
Estimation of S-phase duration in *T. brucei* TREU927. (**A,B)** The doubling time (dt) for procyclic forms of *T. brucei* was estimated to be 8.5 h (r2 = 0.9906). This estimate was confirmed taking the values at exponential phase and using Doubling Time software (http://www.doubling-time.com). Error bars indicate SD of three independent experiments. (**C)** DAPI-labeled parasites (2K2N) were used to measure the percentage of parasites in cytokinesis, which was estimated to be 6.99 ± 1.13% (n = 358). Error bars represent SD. The scale bars for the fluorescence images correspond to 2 µm. This value was used in Eq. 1 (19) to estimate the cytokinesis-phase duration. (**D)** To estimate the duration of the G2 + M phases, EdU was added to the culture, and parasites were continuously collected every 15 min until parasites containing two EdU-labeled nuclei in the same cell (cytokinesis) were observed. This pattern was observed after 2 h. This assay was carried out in triplicate, and in all replicates, we found a parasite containing two EdU-labeled nuclei at the same time. Scale bar = 2 µm. (**E)** EdU-labeled parasites (after 1 h EdU pulse) were used to estimate the percentage of parasites able to uptake this thymidine analog (39 ± 2.7%). Error bars represent SD. Scale bar = 2 µm. This value was used in Eq. 2 to estimate the S-phase duration. These assays were carried out in biological triplicate (n = 358 parasites). (**F)** New estimates for G1- and S-phase duration. ccu means cell cycle unit, where one unit corresponds to the specific doubling time for each strain.

First, we performed growth curves to estimate the doubling time (Fig. 1A,B), which was used in Eqs. 1 and 2 (see Materials and methods)^19,20^. In addition to the doubling time, we also estimated the percentage of parasites performing cytokinesis (C), which was measured through the morphology of the nuclei and kinetoplasts stained with DAPI and differential interference contrast (DIC) (Fig. 1C). *T. brucei* procyclic forms with 2N2K configuration were used to estimate the duration of C phase using Eq. 1^19^, estimated as 0.82 h or 0.096 cell cycle unit (ccu). We found 6.99 ± 1.13% 2N2K parasites from an assay carried out in biological triplicate (Fig. 1C). To estimate the duration of the nuclear G2 + M phases, *T. brucei* cells were continuously collected every 15 min in the presence of EdU until parasites containing two EdU-labeled nuclei (2N2K) in the same cell (C) were observed (Fig. 1D). This pattern was first detected after 2 h, indicating that cells at the end of S phase required 2 h to proceed through G2 and M phases (Fig. 1D). This assay was carried out in triplicate and in all replicates, we found a parasite containing two EdU-labeled nuclei at the same time indicated. EdU-labeled parasites 1 h after an EdU pulse indicated the proportion of parasites able to replicate DNA (39 ± 2.7%) (Fig. 1E). Using this proportion and the estimated duration of the G2 + M + C phases, we were able to calculate the duration of S phase using Eq. 2^20^. S phase was estimated to be 2.31 h or 0.272 ccu (Fig. 1F). The duration of G1 phase was calculated as the difference between the sum of the duration of the other phases (S + G2 + M + C) and the doubling time. Thus, G1 phase duration was estimated to be 3.37 h or 0.397 ccu (Fig. 1F).

These new estimates values for the duration of G1 and S phase in *T. brucei* TREU927 share close similarities to those obtained for *T. brucei* Lister strain 427 (G1 = 3.37 h, S = 2.31 h for TREU927 and G1 = 3.5 h, S = 2.15 h for Lister 427)^15^, suggesting that the different strains of *T. brucei* analyzed do not have significant differences in cell cycle lengths.

### The constitutive origins mapped in *T. brucei* are not enough to accomplish complete DNA replication within the S-phase duration

Using the newly estimated S-phase duration, we estimated the minimum number of origins (*mo*) on each megabase chromosome of *T. brucei* through the application of Eq. 3 (see Materials and methods). During this estimation, we used two different values for the replication rate (*v* = 3.7 kb.min^−1^ and *v* = 1.84 kb.min^−1^) reported by different studies^3,17^, which resulted in two different values for *mo* (Table 1). These two values for *v* were calculated using a DNA combing technique, but each had a different approach^3,17^. Interestingly, another study developed in *T. brucei* mapped the position of activated origins by an MFA-seq technique, which matched ORC binding sites identified by ChIP-seq.^16^. This colocalization strengthens the assertion that these activated origins are indeed true origins and not possible products of other processes that generate DNA synthesis, such as DNA repair. However, the same study identified many more ORC binding sites than activated origins^16^, which can be easily explained by the fact that MFA-seq (genome-wide analysis) is a technique that provides a result based on population analysis. Thus, the set of activated origins that matches with ORC binding sites can be classified as constitutive, and the remaining ORC binding sites are potential sites for the firing of non-constitutive (backup) origins, whether flexible or dormant^6,14^.

**Table 1:** Comparison between the minimum numbers of origins (estimated using two different *v* values) and the constitutive origins detected by MFA-seq technique.

*T. brucei*				
Chromosome	estimated chromosome size	minimum number of origins estimated using v = 3.7 kb.min^−1 ^^17^	minimum number of origins estimated using v = 1.84 kb.min^−1 ^^3^	constitutive origins mapped by MFA-seq^16^
*I*	1064,672 kb	2	3	2
*II*	1193,948 kb	2	3	3
*III*	1653,225 kb	2	4	3
*IV*	1590,432 kb	2	4	3
*V*	1802,303 kb	2	4	3
*VI*	1618,915 kb	2	4	2
*VII*	2205,233 kb	3	5	2
*VIII*	2481,19 kb	3	5	6
*IX*	3542,885 kb	4	7	4
*X*	4144,375 kb	5	9	6
*XI*	5223,313 kb	6	11	8

S-phase duration = 138.6 min (current study).

**Figure 2:**
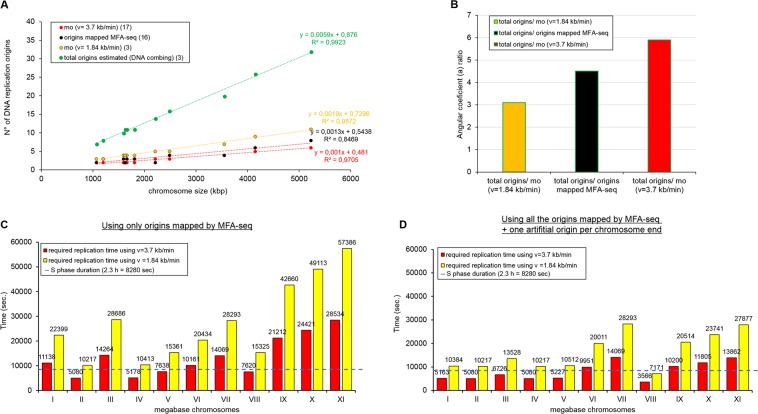
The constitutive origins mapped in *T. brucei* are not enough to accomplish the complete DNA replication within S-phase duration. (**A**) Graph showing positive correlations between chromosome length and the number of replication origins estimated by DNA combing (total origins) (green dots), number of origins estimated by MFA-seq (black dots), minimum origins (*mo*) estimated using *v* = 3.7 kb.min^−1^ (red dots), and *mo* estimated using *v* = 1.84 kb.min^−1^ (yellow dots). The trend lines for all groups, as well as the equations, are shown. (**B)** Angular coefficient ratios between total origins and *mo* using *v* = 1.84 kb.min^−1^ (yellow bar = 3.1), total origins and origins estimated by MFA-seq (black bar = 4.5), and total origins and *mo* using *v* = 3.7 kb.min^−1^ (red bar = 5.9). (**C)** Minimum time required for each *T. brucei* chromosome to complete DNA replication according to the positions of the origins mapped by MFA-seq (16), using two different values for the replication rate: *v* = 3.7 kb.min^−1^ (red) or *v* = 1.84 kb.min^−1^ (yellow). The dashed line represents the estimated S-phase duration reported in this study. (**D)** To resolve the bias generated by the possible presence of origins hidden in subtelomeric/telomeric regions, we repeated the assay shown in C by adding an artificial origin per chromosome end. Of note, each origin was localized 50 kb upstream of the chromosome end.

Comparing the two *mo* numbers estimated for each chromosome using Eq. 3 with the number of constitutive origins, we easily notice that the *mo* estimated with *v* = 3.7 kb.min^−1^ shares close similarities with the number of constitutive origins (Table 1 and Fig. 2A). However, the *mo* values estimated with *v* = 1.84 kb.min^−1^ are slightly above those represented by constitutive origins (Table 1 and Fig. 2A), which makes us wonder which of the two *v* values would allow the constitutive origins complete replication within the S-phase duration. Interesting, in the same study in which *v* = 1.84 kb.min^−1^ was estimated, the authors suggested an average inter-origin distance (IOD) of 148.8 kb, *i.e*., one origin per ~148.8 kb. If we extrapolate this to all eleven chromosomes, we find an average number of origins per chromosome that is 3.1 times higher than the *mo* estimated with *v* = 1.84 kb.min^−1^, 4.5 times higher than the constitutive origins estimated by MFA-seq, and 5.9 times higher than the *mo* estimated with *v* = 3.7 kb.min^−1^ (Fig. 2B). Although these data suggest that *T. brucei* uses a very large number of total origins to complete DNA replication, the average IOD estimated by DNA combing has a bias that impairs its reliability: how do we know if the molecules used for the calculation represent the entire genome? In other words, since the quantification of molecules is carried out in a population, it is possible that several origins from the same chromosomal fragment are measured, which would introduce a bias into the analysis. In this way, we decided at first not to use the average IOD as a priori information in our simulations, even though it corroborates our results. Of note, *T. brucei* naturally shows many more than 11 chromosomes^21^, but only the sequences of the megabase-sized chromosomes are available in the TriTrypDB database (www.tritrypdb.org). Thus, all analyses performed on *T. brucei* considered only these chromosomes, whose encompass the majority of *T. brucei* genes (~9068 predicted genes)^22^.

To determine if the given set of constitutive origins from *T. brucei* can complete the replication of each chromosome within the S-phase duration, we developed another formula (Eq. 4). With this equation, we can calculate the minimum required time for DNA replication using only the set of constitutive origins under two conditions: *v* = 3.7 kb.min^−1^ or *v* = 1.84 kb.min^−1^ (see Materials and methods). When we used the fastest of these two estimations for *v* (3.7 kb.min^−1^), DNA replication was not completed within S-phase duration since only four out of eleven chromosomes could accomplish DNA replication in this time frame (Fig. 2C – red columns, Table S1). These data may be contradictory since MFA-seq identified more origins than *mo* (Fig. 2A); however, the results are easily justified due to the positions of the mapped origins^16^, which do not allow an ideal configuration to optimize replication. Using *v* = 1.84 kb.min^−1^, the situation was even worse, as none of the eleven chromosomes allowed DNA replication within the S-phase duration (Fig. 2C – yellow columns, Table S1).

It is worth mentioning that our *in silico* analyses used the value for full chromosome lengths available at TriTrypDB. However, the lengths of *in silico* chromosomes may not reflect the real *in vivo* chromosomal lengths in trypanosomatids since the annotation of some regions may not have been carried out with the necessary accuracy and details – for instance, the contigs containing subtelomeric and telomeric repeats^23,24^. Indeed, poorly annotated regions may also affect MFA-seq analysis since the localization of possible origins at subtelomeric/telomeric regions would be impaired. However, this would only be true for origins fired in late S phase, as those fired in early/middle S phase would potentially be detected because the upstream replisomes would have enough time to reach the innermost regions of the chromosomes. A genome-wide analysis of chromosome VI from *T. brucei* provides evidence for this statement^16^. Even so, to address this critical point, we again carried out the simulations shown in Fig. 2C but introduced one artificial origin in each chromosome end (each origin was localized 50 kb upstream of the chromosome end). We proposed to determine if the set of constitutive origins mapped by MFA-seq plus the artificial subtelomeric/telomeric origins could complete replication within the S-phase duration. Surprisingly, it was not possible to complete DNA replication within the duration of S phase in the presence of artificial origins using a replication rate of *v* = 3.7 kb.min^−1^ or *v* = 1.84 kb.min^−1^. Using *v* = 3.7 kb.min^−1^, six out of eleven chromosomes could accomplish DNA replication in the appropriate time (Fig. 2D – red columns, Table S1). Using *v* = 1.84 kb.min^−1^, only one out of the eleven chromosomes could complete replication within the S-phase duration (Fig. 2D – yellow columns, Table S1).

### A mechanistic model of DNA replication considering replication-transcription conflicts can predicts the behavior of origins usage

Considering that trypanosomatids organize the majority of their genes into large polycistronic gene clusters^11,25^, an issue can be raised: could an event related to transcription and replication (*e.g*., replication-transcription conflicts) contribute to the firing of non-constitutive origins, thus increasing the pool of origins used, as previously suggested in other cell types ^6,9,26^? To investigate the possible impact of replication-transcription conflicts on origin firing, we designed a stochastic dynamic model that constrained the availability of resources by establishing a limit for the number of available replisomes during the simulation (parameter F), as previously done in a similar model^27^. Of note, in human cells, the role played by the parameter F in our model is performed by CDC45 protein^28^. However, we do not know if CDC45 ortholog plays the same role in trypanosomatids. Also, this model has a probability of replication origin firing modulated by the probability landscape reported in Fig. S1 (blue lines).

**Figure 3:**
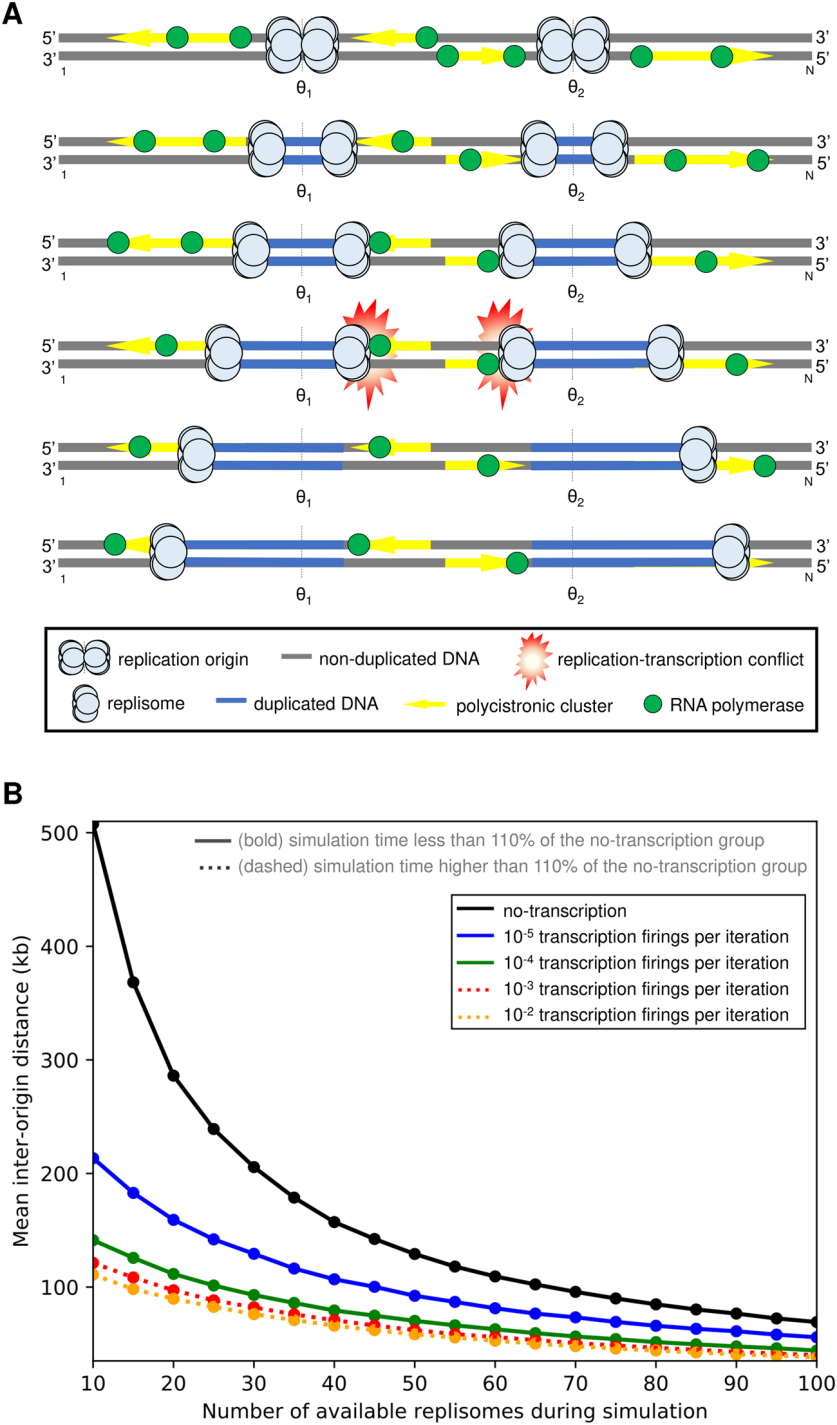
A mechanistic model of DNA replication considering replication-transcription conflicts predicts an increase in the number of origins activated to keep robustness in S-phase duration. (**A)** Example of replication-transcription conflicts in *T. brucei*. When replication and transcription are simulated at the same time, if there is a *head-to-head* collision (replication-transcription conflicts), then the replication fork collapses, leading to an unbinding of its respective replisome and the involved RNAP. (**B)** Results of simulations with a stochastic dynamic model show the mean inter-origin distance (which is inversely proportional to the activation of origins) as a function of the transcription frequency and the number of available replisomes during a simulation (parameter F). The black line represents sets of simulations without the presence of constitutive transcription. The remaining bold lines (blue and green) represent simulations with constitutive transcription whose adopted frequencies (10^−5^ and 10^−4^ RNAP firings per iteration, respectively) result, for each F value, in a mean simulation time less than 110% of the no-transcription group. On the other hand, the dashed lines (red and orange) are simulations whose adopted frequencies (10^−3^ and 10^−2^ RNAP firings per iteration, respectively) incur in a mean simulation time higher than 110% of the no-transcription group.

The model is composed of a set of binary vectors, one vector per chromosome (Fig. S2). The dimension of each vector was equal to the number of base pairs in that chromosome; therefore, each dimension consisted of a binary flag that assigns whether its corresponding base pair was replicated or not. In this model, we assume the presence of non-constitutive origins with a passive mechanism (*i.e*., origins that fire stochastically). To this end, we modeled the replication process for each chromosome as a Markov chain whose state transition was defined as a function of two parameters: the probability of an origin firing when it is selected at base i in chromosome j (p(i, j)), and the maximum number of replisomes activated simultaneously (*F*). p(i, j) was estimated through the linear transformation of MFA-seq data^16^; this transformation is depicted by the blue lines in Fig. S1. When a given replication origin is fired, it spawns two replication forks that move in opposite directions (Fig. S3). DNA replication progress was accomplished by updating, at each iteration, the binary vectors that represent the replication state of all chromosomes. We also included in the model constitutive transcription, *i.e*., transcription whose levels (frequency) remain fairly constant despite the cellular condition. The average transcription rate of RNA polymerase (RNAP) was set to be the same as the replisome replication rate (*v*). Transcription was modeled with the periodic firing of RNAP at the beginning of each polycistronic region (Fig. S4). We tested five possibilities for the RNAP firing frequency: 1) no RNAP firing at all; 2–5) one RNAP firing every 100000, 10000, 1000 or 100 iterations of the simulation. The collisions between replisome and RNAP arise when we simulate together previously described replication and transcription dynamics in the same model (Fig. 3A). In our simulations, we focused our attention on *head-to-head* collisions, in which the replisome and RNAP come from opposite directions. Once we assume that the transcription velocity is equal to the replication rate, there are no *head-to-tail* collisions, *i.e*., there is no possibility of an RNAP colliding behind a replisome or vice-versa. The outcome of *head-to-head* collisions was designed following the scenario modeled by Lin and Pasero (2012)^29^: if one such event occurs, then there is a fork collapse, and the replisome is released from the chromosome. We carried out several sets of Monte Carlo simulations (270 simulations per F value and RNAP firing period). The obtained results were then averaged (Table S2).

Initially, for different values of F (number of available replisomes), we evaluated the model dynamics without the presence of transcription. In these simulations, we observed that there was a decrease in the mean IOD as we increased the number of available replisomes (black line in Fig. 3B, Table S2), with mean IOD values ranging from 507.6 kb (F = 10) to 69.2 (F = 100). Of note, the mean IOD calculated using putative constitutive origins was equal to 631.4 kb, *i.e*., all sets of results provided by the dynamic model were below this upper bound for the mean IOD. Interestingly, for F = 45, the mean IOD was 142.3 kb, which was almost the same as the 148.8 kb suggested in a previous study^3^.

Next, we evaluated the impact of increasing frequencies of constitutive transcription on replication-transcription conflicts and S-phase duration. These simulations indicated a reduction in the mean IOD (which is inversely proportional to the number of replication initiations) in response to increasing frequencies of constitutive transcription and values of the parameter (Fig. 3B, blue, green, red, and orange lines; Table S2). Even simulations with a low transcription frequency (e.g., an RNAP firing every 100000 simulation iterations) led to a reduction in the mean IOD relative to simulations without transcription (Fig. 3B, compare black and blue lines). Additionally, the model displayed robustness in S-phase duration for moderate amounts of transcription: for all tested RNAP firing periods and all F values, only simulations with ≤10^−3^ transcription firing per iteration demanded more than 110% of the mean number of iterations required by no-transcription simulations (Fig. 3B, red and orange lines, Table S2).

Thus, under standard conditions, replication-transcription conflicts might be responsible for a decrease in the estimated mean IOD, making the parasite fire a pool of origins higher than the minimum required to complete S phase. To verify the reliability of this simulation, we decided to validate these predictions experimentally, starting by ascertaining the presence of transcription during S phase.

### Transcription in *T. brucei* is carried out throughout the cell cycle and its short-term inhibition does not impair DNA replication

**Figure 4:**
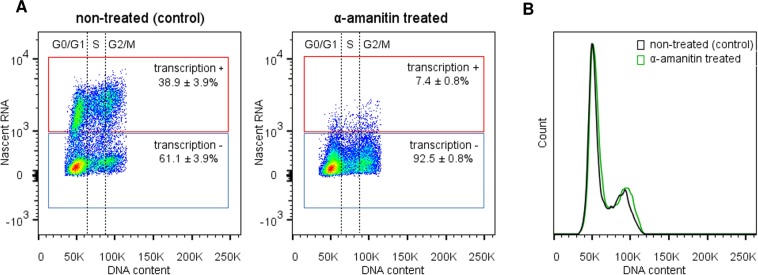
Transcription landscape throughout the *T. brucei* cell cycle. (**A)** Density plot showing the distribution of the population according to DNA content and nascent RNA synthesis. Left – Non-treated (control) population showing transcriptional activity (nascent RNA) = 38.9 ± 3.9% (red square). Right – After transcription inhibition using α-amanitin, nascent RNA decreased to 7.4 ± 0.8% (red square). The cell cycle phases (G0/G1, S, and G2/M) are indicated. Red square – transcription-positive, blue square – transcription-negative. (**B)** Histograms show virtually unchanged DNA content profiles for the control (black line) and α-amanitin treated groups (green line). These assays were carried out in biological triplicate, and 20,000 parasites (n = 20,000) were counted in each analysis.

To check if *T. brucei* was able to limit its transcription during replication to avoid potential collisions, the parasites were subjected to an *in vitro* transcription assay (called nuclear run-on), coupled with DNA content analysis using DAPI. This approach enabled us to analyze the transcription of nascent RNAs during different cell cycle phases. In this way, we analyzed transcriptional activity according to the DNA content profile in a wild type population of *T. brucei* (control) (Fig. 4A – left). We observed transcription activity during all phases of the cell cycle (G0/G1, S, and G2/M), including during replication (Fig. 4A, transcription+). To validate this data, we repeat the assay performing a pre-treatment of the parasites with α-amanitin, an irreversible transcription inhibitor. The α-amanitin treatment caused a drastic decrease in the signal observed, from 38.9 ± 3.9% to 7.4 ± 0.8% (Fig. 4A – to compare left and right graphs). This result validates our analysis and confirms that the fluorescent signal measured was indeed from nascent RNAs. Furthermore, Fig. 4B shows the unchanged DNA content profiles of both analyzed groups, demonstrating that the short-term α-amanitin treatment used in this study was enough to inhibit transcription without impairing DNA replication.

### Endogenous foci of γH2A and R-loops are dependent on the transcription activity

Recent studies have been demonstrating a correlation among DNA lesions, R-loops, and replication-transcription conflicts^8,30,31^. To investigate these features in *T. brucei*, we used immunofluorescence assays (IFA) to measure the fluorescent levels of endogenous DNA lesions and R-loops in different cell cycle phases. Importantly, the morphological distinction of the cell cycle phases was performed based on DAPI-stained nuclei/kinetoplasts and DIC, allowing us to differentiate cells with nuclei in G1/early S, late S/G2, mitosis, and cytokinesis^32–35^. To verify potential endogenous levels of DNA lesions, we measured the fluorescence intensity of the histone γH2A, which is commonly used as a DNA break biomarker^33,36,37^. It is worth mentioning that in mammals, the phosphorylation resulting from DNA lesions happens in the variant histone H2AX, which generates γH2AX. However, the presence of this variant histone has not been reported in trypanosomatids or yeast^33,38^. Several studies indicate that the canonical phosphorylated histone (γH2A) plays the role of γH2AX in these organisms, *i.e*., increasing *in vivo* in response to DNA lesions^33,39,40^.

**Figure 5:**
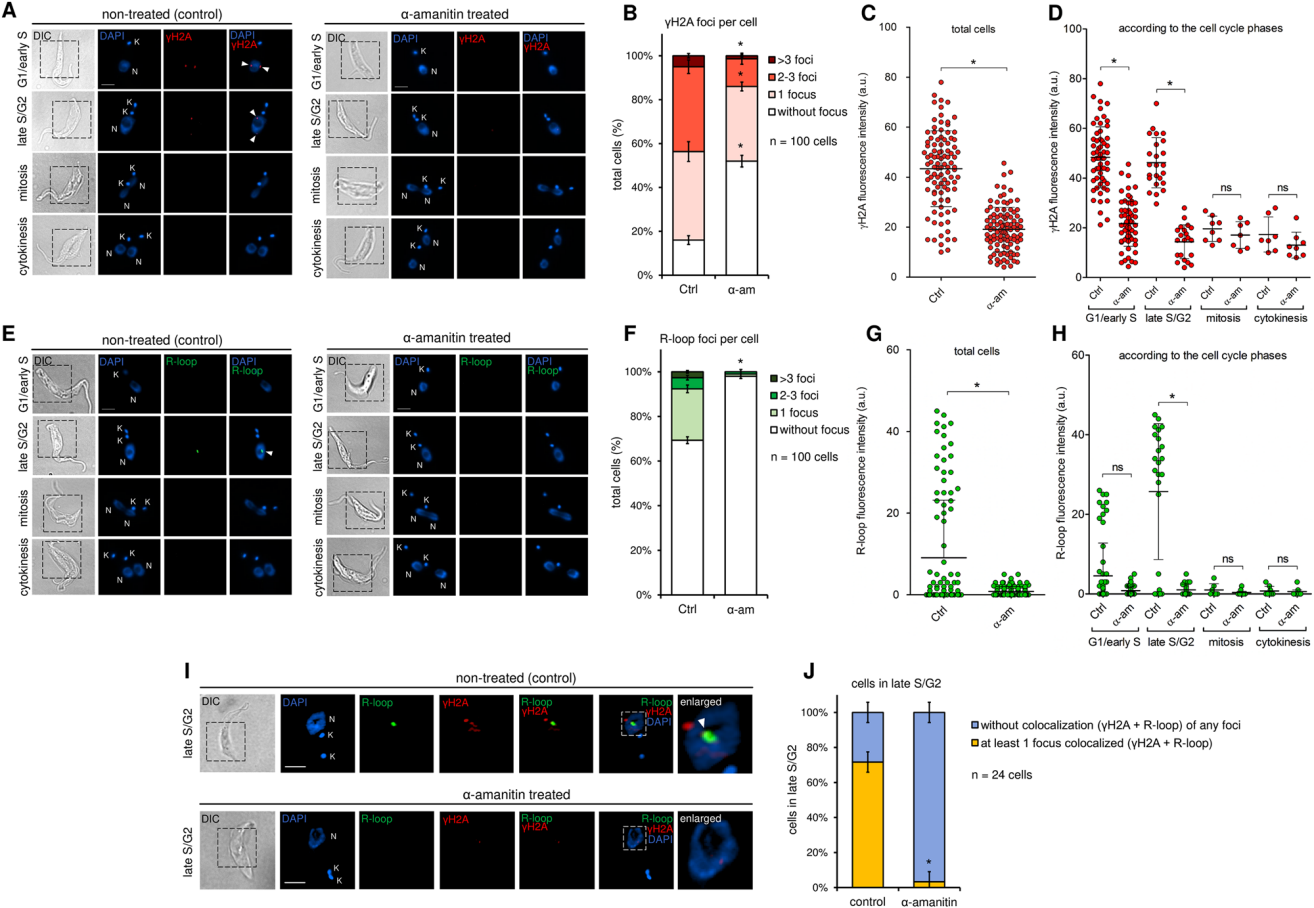
DNA lesions and R-loops are dependent on the transcription activity and partial colocalize at late S/G2 phases. (**A)** Left – The endogenous γH2A foci shown by nontreated (control) parasites suggest the presence of DNA lesions mainly during G1/early S and late S/G2. Right – γH2A foci decreased after transcription inhibition (α-amanitin treated). (**B)** Graph showing the number of γH2A foci per cell before and after α-amanitin treatment. (**C**) Graph showing the γH2A fluorescence intensity (red) per cell in total cells and (**D)** according to the cell cycle phase analyzed. Errors bars indicate SD. The difference observed was statistically significant using Student’s t-test (*p < 0.001) for a biological triplicate assay (n = 100). (**E)** Left – The control parasites showed endogenous R-loops foci predominantly during late S/G2. Right – After transcription inhibition, these R-loop foci decreased. (**F)** Graph showing the number of R-loop foci per cell before and after α-amanitin treatment. (**G)** Graph showing the R-loop fluorescence intensity (green) per cell in total cells and (**H)** according to the cell cycle phase analyzed. Errors bars indicate SD. The difference observed was statistically significant using Student’s t-test (*p < 0.01) for a biological triplicate assay (n = 100). (**I)** Representative confocal images show partial colocalization (white triangle) between γH2A (red) and R-loop (green) during late S/G2 in the control group. After transcription inhibition, this partial colocalization disappeared, as expected. (**J)** Bar graph shows 69.9 ± 3.35% of parasites that are in late S/G2 phase show at least 1 focus colocalized (γH2A + R-loop). After transcription inhibition, this value fell to 1.1 ± 1.9%. Yellow bar – at least one focus colocalized (γH2A + R-loop), blue bar – without colocalization of any foci (γH2A + R-loop). Errors bars indicate SD. The difference observed was statistically significant using Student’s t-test (*p < 0.01) for a biological triplicate assay (n = 24 parasites). Using Pearson’s correlation coefficient, we obtained r = 0.5, suggesting a moderate correlation.

We observed that *T. brucei* shows endogenous levels of DNA lesions represented by a few γH2A fluorescent foci (red), predominantly throughout the G1, S, and G2 phases (Fig. 5A,D). These foci became rare during the mitosis and cytokinesis phases (Fig. 5A,D). This behavior can be explained by the fact that, when DNA lesions occur (probably during the S phase), their repair takes place predominantly in the late S or G2 phases via the homologous recombination (HR) pathway^40,41^. When we inhibit transcription via treatment with α-amanitin, we observed a decrease in γH2A fluorescence intensity during G1/early S and late S/G2 (Fig. 5A,D). Analyzing the same data considering the total number of cells, we observed a significant decrease in γH2A fluorescence intensity, as well as, a decrease in the number of foci per cell (Fig. 5B,C).

An essential and pertinent observation that may be raised relates to the immediate phosphorylation of H2A and its dependence on transcription, *i.e*., how do we know if the immediate decrease in γH2A fluorescence intensity represents a bias due to the absence of transcription? To address this critical point, which is inherent to this assay, we induced DSBs using 50 Gy of ionizing radiation (IR) in the presence or absence of α-amanitin (Fig. S5). We did not detect a decrease in γH2A fluorescence intensity after inducing DSBs in cells with transcription inhibited. Thus, we might state that the immediate phosphorylation of H2A did not depend directly on nascent RNAs that were inhibited during the treatment (Fig. S5). Together, these results suggest that transcription-dependent DNA lesions are generated endogenously in *T. brucei* predominantly during S phase and remain until G2, when the parasite repairs the damage and γH2A phosphorylation is removed (Fig. 5A,D). Of note, this pattern was not detected in parasites that were treated with 50 Gy because IR reaches the parasites in all cell cycle phases instantaneously, generating DSBs in all of them (Fig. S5).

To verify potential R-loop foci in *T. brucei*, we used a specific antibody (S9.6) that recognizes DNA:RNA hybrids^42–44^ (see Material and methods). R-loops are triple-stranded nucleic acid structures composed of a DNA:RNA hybrid and a single-stranded DNA (ssDNA), and their generation is related to various factors, among them replication-transcription collisions^8,30,31,45^. Like γH2A, *T. brucei* shows endogenous levels of R-loops throughout the S and G2 phases, which become rare during mitosis and cytokinesis (Fig. 5E,H). Thus, we may state that probably R-loops are resolved before cells enter mitosis. Moreover, when transcription is inhibited, we observed a decrease in R-loop fluorescent foci (Fig. 5E–H). When we analyze the same data considering the total number of cells, we observed a significant decrease in R-loops fluorescence intensity, as well as a decrease in the number of foci per cell after transcription inhibition (Fig. 5F,G).

### γH2A and R-loop foci partial colocalize in late S/G2 phase

To obtain evidence to determine if DNA lesions observed are associated with the replication-transcription conflicts, we asked whether γH2A and R-loops colocalize since both are observed predominantly in the same cell cycles phases (from S to G2) and are transcription-dependent. For this, we performed IFA colocalization assays using confocal microscopy. We observed partial colocalization (indicated by the white triangle) only in late S/G2 cells, represented by cells with a 1N2K configuration (Fig. 5I). This finding indicates that γH2A accumulates at R-loop-enriched foci. From cells in late S/G2 analyzed (n = 24), 69.9 ± 3.35% showed at least one focus partial colocalized, while the remaining cells do not show colocalization of any foci or do not show γH2A /R-loop foci. When we inhibited transcription via treatment with α-amanitin, the partial colocalization pretty much disappears (3.3 ± 5.8%), as expected (Fig. 5J). This result suggests that besides being transcription-dependent, γH2A and R-loops foci are generated from the same location, perhaps a region prone to replication-transcription conflicts.

### R-loops contribute to the generation of the endogenous γH2A foci observed

**Figure 6:**
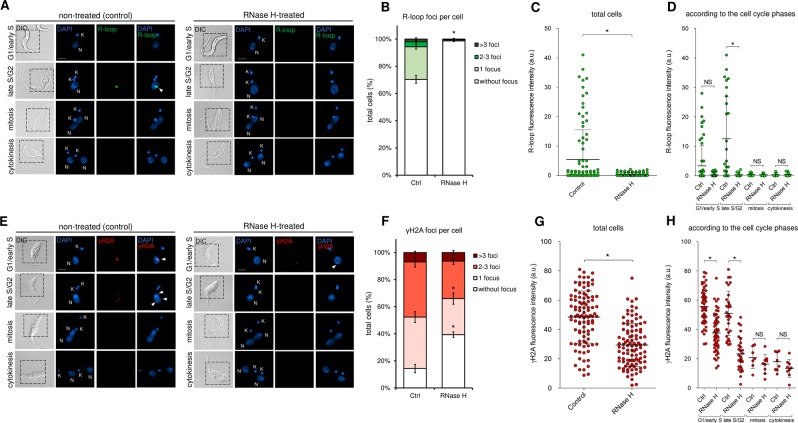
R-loops contributes to the generation of DNA lesions. (**A**) RNase H treatment resolves R-loops. Just like results showed on Fig. 5E, the non-treated (control) parasites showed basal levels of R-loops only during late S/G2 (green). After RNase H treatment, the basal levels of fluorescence intensity, as well as the number of R-loop foci per cell disappear, which confirm the specificity of the antibody used. (**B)** Graph showing the number of R-loop foci per cell before and after RNase H treatment. (**C)** Graph showing the R-loop fluorescence intensity (green) per cell in total cells and (**D)** according to the cell cycle phase analyzed, before and after RNase treatment. (**E)** After RNase treatment, the endogenous levels of γH2A fluorescence intensity, as well as the number of foci per cell, decrease significantly, which suggests that R-loops contributes to the DNA lesions observed. (**F)** Graph showing the number of γH2A foci per cell before and after RNase H treatment. (**G)** Graph showing the γH2A fluorescence intensity (red) per cell in total cells and (**H)** according to the cell cycle phase analyzed, before and after RNase treatment. Error bars indicate SD. The differences observed were statistically significant using the Student’s t-test (*p < 0.05).

To check if R-loops are associated with the DNA lesions observed, we performed IFA using antibodies against R-loops and γH2A after RNase H (a ribonuclease that catalyzes the cleavage of RNA from a DNA:RNA substrate) treatment. We observed that RNase H was able to decrease R-loop fluorescent intensity (Fig. 6A,C,D), as well as R-loop foci per cell (Fig. 6B) in a way similar to α-amanitin treatment (to compare Fig. 6A,C with Fig. 5E,G). When we analyze the γH2A signals (DNA lesions) after RNase H treatment, we observed a significant decrease in fluorescence intensity predominantly in S/G2 phases (Fig. 6E,G,H), as well as a decrease in the number of foci per cell after RNase H treatment (Fig. 6E,F). Together, these findings suggest that R-loops is contributing to the endogenous DNA lesions presented by *T. brucei*.

### *T. brucei* activates fewer replication origins and exhibits a higher average replication rate after transcription inhibition

**Figure 7:**
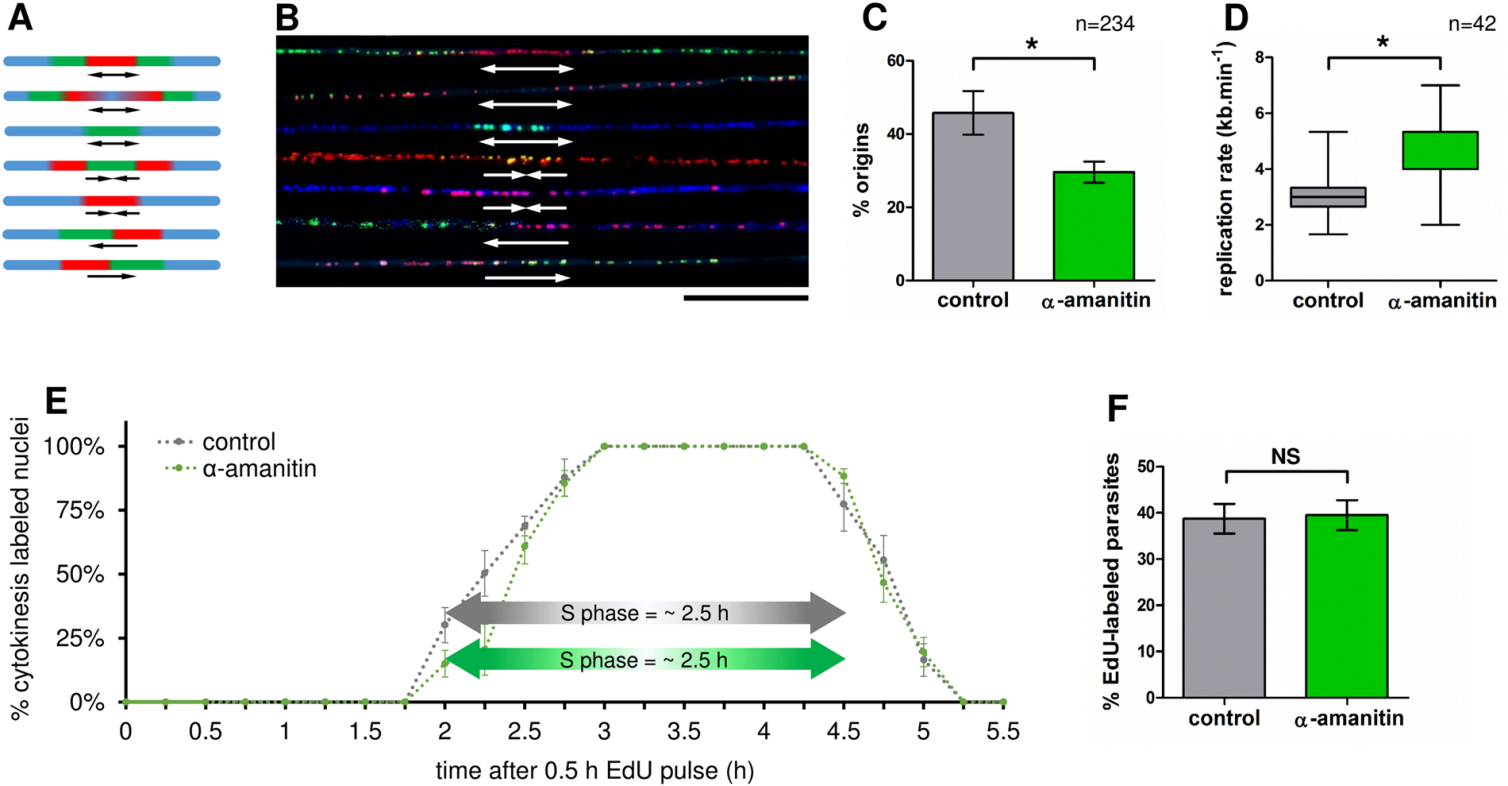
Transcription contributes to the backup-origins firing helping to maintain robustness in S-phase duration. (**A)** Scheme showing the possible DNA fibers-patterns we looked for during our analysis. (**B)** Representative images of the DNA fibers analyzed after a random capture of several image fields. From top to bottom, the fibers represent: origin, origin, origin, termination, termination, replication fork, and replication fork. The arrows represent the fork direction. Bar = 20 μm. **(C)** Bar graph showing that the percentage of origins measured in control (45.77 ± 3.4%) relative to α-amanitin treated group (29.6 ± 1.6%). Error bars indicate SD. The differences observed were statistically significant using the Student’s t-test (*p < 0.05) for a biological triplicate assay (n = 234). (**D)** Using the formula $(\mathop{\sum }\limits_{1}^{42}length/42)/time\,of\,CldU\,pulse$, we compared the replication rate in control (3.06 ± 0.21 kb.min^−1^) and α-amanitin treated group (4.76 ± 0.27 kb.min^−1^). Error bars indicate SD. The differences observed were statistically significant using the Student’s t-test (*p < 0.05) for a biological triplicate assay (n = 42). **(E)** To estimate the S-phase duration after transcription inhibition, we performed a 30-min EdU pulse and quantified the percentage of cytokinesis-labeled nuclei every 15 min. The bars represent the SD from an assay carried out in biological triplicate (n = 20 cells for each time point analyzed, totaling n = 460 cells). (**F)** Bar graph showing the percentage of parasites able to uptake EdU after a 30-min pulse: 38.7 ± 3.2% for control and 39.5 ± 3.2% for the α-amanitin-treated group. The difference observed was not statistically significant using Student’s t-test (NS = not-significant) for a biological triplicate assay (n = 569 cells).

To experimentally measure the percentage of origins fired and the replication rate after transcription inhibition, we used the DNA combing technique. This approach allows the visualization of replication origins in replicated DNA molecules stretched on coverslips via the subsequent incorporation (in a short-term period) of halogenated thymidine analogs: IdU and CldU. Once incorporated into DNA, these thymidine analogs can be detected by specific antibodies in a way similar to BrdU approach^15^, allowing the distinction of each of these analogs through subsequent reactions using secondary fluorescent antibodies (red for IdU and green for CldU). The DNA molecules were also labeled with anti-ssDNA antibody (blue). With this technique, it is possible to detect the origin and termination regions, as well as fork movement. Also, the replication rate is calculated by dividing the length of CldU-incorporated DNA (in kb, using the stretching factor 1 µm = 2 kb, provided by Genomic Vision) by the duration of the CldU pulse (30 min). Of note, the CldU-incorporated DNA used must be between an IdU-incorporated DNA and a DNA signal to ensure complete incorporation during the period of CldU pulse. More details about this approach can be found in the Material and methods section, and in other studies of our group^17,46^. Figure 7A shows a scheme with the DNA fibers-patterns we looked for during our analysis and Fig. 7B shows representative images of these DNA fibers that were analyzed in our assay after a random capture of images. The arrows represent the replication fork direction. From 234 DNA fibers (118 in control and 116 in α-amanitin), we observed 45.77 ± 3.4% origins in control and 29.6 ± 1.6% in the α-amanitin-treated group (Fig. 7C). Furthermore, the average replication rate increased from 3.06 ± 0.21 kb.min^−1^ (control) to 4.76 ± 0.27 kb.min^−1^ after transcription inhibition (α-amanitin-treated) (Fig. 7D).

Besides corroborating our mechanistic computational simulations carried out previously (compare these results with those in Fig. 3B and Table S2), this finding suggests that with decreased replication impairments (transcription inhibition) fewer origins are activated, probably because there is no need to fire backup origins. Thus, with fewer impairments, the replisome can synthesize a larger segment of DNA contributing to the measurement of a higher average replication rate. In summary, this finding points to the contribution of the transcription machinery in the firing of origins, possibly as a result of replication-transcription conflicts.

### S-phase duration remains the same after transcription inhibition

To quickly measure S-phase length during transcription inhibition, avoiding possible transcription-dependent interference, we decided to use an approach that does not rely on the measurement of other phases of the cell cycle, such as those performed in Fig. 1. To this end, we followed an established assay in trypanosomatids^15^, which directly yields kinetic determinations of S phase through the measurement of cytokinesis (2N2K)-labeled nuclei. In this assay, we performed a 15-min EdU pulse to subsequently measure the cytokinesis of EdU-labeled nuclei continuously, until the EdU signal disappears. There was an initial period in which cytokinesis was not labeled, and this period finished with the onset of cytokinesis-labeled nuclei that sharply increased up to 100% and stayed at this level for a defined period, yielding estimates of S-phase length: ~2.5 h for both control and α-amanitin-treated groups (Fig. 7E). Both curves were practically coincident, which means that S-phase duration was not affected by transcription inhibition (Fig. 7E). Of note, during these kinetic experiments, the parasite populations grew exponentially and in a steady-state manner. Despite the exponential growth of the populations, labeling indexes displayed approximate linear increases since the measures were carried out in a period shorter than the doubling-time (dt) for *T. brucei*. Besides, the capacity of the parasites for EdU uptake after α-amanitin treatment remained largely the same (Fig. 7F). This finding corroborates those obtained in Fig. 4, demonstrating that short-term transcription inhibition does not affect DNA replication.

## Discussion

The duration of S phase in *T. brucei* estimated using EdU (Fig. 1) allowed us to estimate the minimum number of origins (*mo*) required to replicate an entire chromosome during S phase. After comparing the *mo* values estimated using the two different replication rates available in the literature (*v* = 3.7 kb.min^−1 ^^17^; *v* = 1.84 kb.min^−1 ^^3^) with the constitutive origins mapped by MFA-seq, we asked if these origins (the constitutive ones) would be sufficient to accomplish DNA replication within the S-phase duration (Fig. 2A,B). Through a stochastic computational model, we determined that these origins are not enough to accomplish complete DNA replication within the S-phase duration, even in the presence of artificial subtelomeric/telomeric origins (Fig. 2C,D). This data demonstrates that the positioning of constitutive origins does not permit the replication of all chromosomes within the S-phase duration. A parsimonious hypothesis that could satisfactorily explain this peculiarity is the activation of non-constitutive (backup) origins to assist in the completion of replication. These origins may be flexible or dormant, although the presence of both is not mutually exclusive, *i.e*., the presence of replication stress that activates dormant origins does not exclude the possibility that the flexible origins are also activated in a stochastic manner. One piece of evidence supporting this hypothesis is that the MFA-seq data found many more ORC binding sites than replication peaks, suggesting the presence of non-constitutive origins in *T. brucei*^16^. In addition, a study that used a DNA combing approach, as previously mentioned^17^, also suggested the presence of backup origins in this parasite.

Based on these features and considering that the S-phase duration estimated previously is robust, as proposed for other cell types^27,47^, simulations with a stochastic computational model suggest that the presence of replication-transcription conflicts might lead to an increase in origin firing, which in turn helps to maintain a robustness in S-phase duration (Fig. 3, Table S2). Together, these simulations suggest the presence of a mechanism responsible for a decrease in the mean IOD (inter-origin distance) estimated under standard conditions, making the parasites fire a pool of origins greater than the minimum required to complete S phase. As trypanosomatids organize most of their genes into large polycistronic gene clusters, we hypothesized that some event related to transcription, which would generate replication stress, could contribute significantly to the activation of backup origins. Our computational model demonstrated that this hypothesis is fully possible since transcription during S phase directly impacts the number of origins required to complete replication in the estimated time frame (Fig. 3, Table S2). As *T. brucei* does not limit transcription during S phase (Fig. 4A), both replisome and RNAP may operate concomitantly in the same region, making it inevitable that these two processes interfere with each other, generating collisions. The consequences of these collisions are often accompanied by R-loops, DNA lesions, recombination or, in some cases, cell death^8,30,31,48–51^.

We provide evidence here that the endogenous levels of DNA lesions shown by *T. brucei* can be a consequence of replication-transcription conflicts through the generation of R-loops (Figs. 5 and 6). DNA lesions were measured indirectly by the presence of γH2A fluorescent foci, which decreased after transcription inhibition (Fig. 5A–C). These findings agree with other studies in trypanosomatids^30,52^ and in cancer cells, which showed endogenous levels of γH2AX at transcription start sites^53,54^. It is important to note that not all γH2A foci decreased after transcription inhibition (Fig. 5A–C), which makes it clear that transcription activity (possibly due to conflicts with replication) contributes to the generation of endogenous DNA lesions, but it is not its sole cause. Thus, we can infer that DNA damage does not appear to be a great obstacle for *T. brucei* to continue to proliferate, as suggested by a recent study^35^. Also, apparently *T. brucei* and others trypanosomatids possess efficient DNA damage repair processes^40,52,55,56^.

Moreover, we detected fluorescent R-loop foci predominantly in late S/G2 (Fig. 5E–H and Fig. 6A–D), which exhibited a partial colocalization with DNA lesions (Fig. 5E–I), corroborating some studies carried out in other cell types^57–59^. Curiously, these R-loop foci were found near the nucleolus, which corresponds to the area that is less heavily stained by DAPI within the nucleus^60^. This can be easily explained by the high levels of transcription carried out by RNAP II in trypanosomatids, which largely takes place in this region^60^. Obviously, this does not imply that R-loops are present only in this region, but since transcription is abundant there, fluorescent foci become more pronounced, while the other possible R-loop sites probably are hidden due to the low sensitivity of the IFA technique. Treatment with exogenous RNase H, an enzyme that resolves these structures^61,62^, confirmed that the foci we observed were indeed R-loops (Fig. 6A). Furthermore, RNase H treatment decreased the DNA lesions measured by γH2A fluorescent foci, suggesting that R-loops is contributing to the endogenous DNA lesions presented by *T. brucei* (Fig. 6B). This finding corroborates a recent study performed with bloodstream forms (BSF) of *T. brucei*, where the loss of *T. brucei* RNase H1 leads to increased levels of replication-associated DNA lesions, predominantly at the chromosome ends^44^. Also, this work showed that the DNA lesions at the chromosome ends are associated with an increase in the VSG switching^44^, an efficient immune evasion strategy used by *T. brucei* BSF^63^. Interestingly, in another study carried out by Briggs (2018)^43^ employing a genome-wide approach, the R-loops showed a conserved localization at centromeres, rRNA genes, and retrotransposon-associated genes, but also showed an abundant localization among coding sequences of the polycistronic transcription units. Although our findings suggest that R-loops contribute to DNA lesions, a genome-wide study using procyclic forms of *T. brucei* is necessary to confirm this correlation and to identify regions prone to replication-transcription conflicts. Furthermore, our assays do not allow us to distinguish if R-loop is a consequence of replication-transcription conflicts or the cause of replication hindering, since R-loops can form stochastically, independent of conflicts, and would be able to impair the replication^64,65^, leading to the assembly of backup origins.

Using a DNA combing approach, we demonstrated that the percentage of activated origins decreases after transcription inhibition, probably because there is no need to fire the backup-origins (Fig. 7C). Also, the average replication rate in the population with transcription inhibited was higher relative to control, which was expected since, with fewer conflicts, the replisome is able to synthesize a larger segment of DNA, contributing to the measurement of a higher average replication rate. Curiously, the replication rates measured in the control and α-amanitin-treated groups (3.06 and 4.76 kb.min^−1^, respectively) (Fig. 7D) were not similar to those used in our previous assays (1.84 and 3.7 kb.min^−1^, see Fig. 2), which means that probably replication rate measurement can vary according to the number and size of intact molecules analyzed^3,17^. Thus, although from this data it is difficult to establish a precise replication rate for wild type cells of *T. brucei*, we can affirm that it is probably between 1.84 and 4.76 kb.min^−1^ and the variation will depend on how many kilobases the replication fork will be able to synthesize without being impaired. Besides, we showed that after transcription inhibition, the S-phase duration remained the same in the period measured (Fig. 7E), which evidence robustness in the S-phase duration. In other words, the impaired replication allows the firing of backup origins to keeps the S-phase duration, as already suggested by our computational model (Fig. 3) and other studies^47,66^.

Intuitively, we can suppose that the newly fired origins would give rise to more collisions with RNAPs from gene clusters adjacent to these origins. However, according to our findings, we did not observe this to occur. We observed in our computational model that the decrease of the mean IOD as a function of increasing levels of transcription frequency converges to a lower bound, which means that newly replication-transcription *head-to-head* conflicts did not occur because of backup origins firing (Fig. 3B). We can speculate on two hypotheses that would help explain this peculiarity either for our *in silico* model and for *in vivo* genomic homeostasis of *T. brucei*. First, as trypanosomatids exhibit a peculiar gene organization into transcription directional gene clusters (DGCs) in opposite directions, the divergent transcription strand-switch regions, which separate two DGCs^67^, would be prone regions to house backup origins, thus avoiding *head-to-head* replication-transcription collisions^68^. Indeed, the presence of origins within DGCs appears to be unusual in trypanosomatids^16,69,70^, which corroborates this hypothesis. However, further assays are needed to find out the position of these backup origins in each mega-chromosome of *T. brucei*. In this regard, the usage of a mathematical model that describes replication failing due to replication fork stalling would be beneficial to support this hypothesis. That model originally applied on five yeast strains^71^, could be adjusted to *T. brucei* and used to make predictions regarding positioning of replication origins and DGCs. The second hypothesis would be the establishment of different replication times for DGCs within the S phase in order to favor replication-transcription *head-to-head* collisions in early S phase, and possible *head-to-tail* collisions (if transcription and replication speeds are different) in late S phase. That is, *head-to-tail* collisions would prevail during backup origins firing. Although it needs to withstand experimental assays to receive credibility, this hypothetical strategy could regulate the generation of replication-associated DNA lesions and consequent genomic instability in regions within DGCs. It is worth mention that different replication times for large transcription units has already been evidenced in model eukaryotes in order to direct, through the action of the transcription machinery, replication-associated DNA lesions and consequent genomic instability^72^. The approach used in this study (BrU-seq)^72^, combined with nanopore DNA sequencing using thymidine analogs^73^, and data from MFA-seq approach^16,70^, is one example of a combined methodological strategy that could provide clues about the behavior of replisome and RNAP during S phase in *T. brucei*.

Another important point worth mentioning is that studies on bacteria and model eukaryotes demonstrate that once stalled, a replication fork can be remodeled, and the replication reactivated at the point of the lesion^74,75^. This event normally generates ssDNA coated with SSB (in prokaryotes) or RPA (in eukaryotes) that induces, respectively, SOS response or S-phase checkpoint^74^. However, S-phase checkpoint in eukaryotes is characterized by the slowing of replication in response to DNA damage^76^ and, in some cases, inhibition of backup origin firing^77^, two features not observed in our findings (Fig. 7C,F). Thus, we hypothesize that *T. brucei* can activate backup origins without trigger a significant S-phase checkpoint in response to replication impairment generated by transcription activity. Nevertheless, further studies are necessary to figure out how trypanosomatids deal with the possible stalled replication forks generated by replication impairment in general.

**Figure 8:**
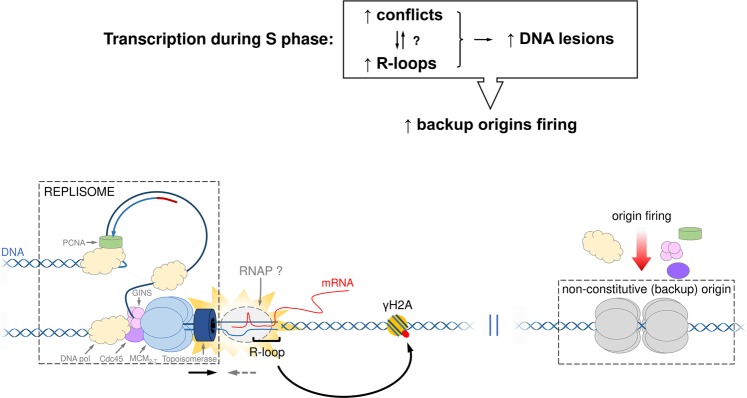
Model showing consequences of the transcription activity during the S phase in *T. brucei*. During the S phase, transcription can generate replication-transcription conflicts and R-loops. Our findings did not allow us to distinguish if R-loops are a consequence of replication-transcription conflicts or the cause of replication hindering, However, according to our data, both conflicts and R-loops contribute to the presence of endogenous DNA lesions (γH2A) and backup-origins firing, helping to maintain robustness in S-phase duration. The backup-origins firing may help to explain the discrepancy observed by us regarding the number of constitutive origins are not enough to allow a complete DNA replication within the S-phase duration (Fig. 2). Further studies are necessary to investigate if the activation of the backup origins occurs in an active (triggered by replication stress) or passive (stochastic) manner.

In conclusion, our findings suggest that the transcription activity during the S phase contributes to the emergence of R-loops and DNA lesions, leading to the firing of non-constitutive (backup) origins that help maintain robustness in S-phase duration. We speculate that this phenomenon occurs due to replication-transcription conflicts, although further assays are necessary to confirm this hypothesis (Fig. 8). The use of an entire pool of origins (constitutive + backup origins) is apparently necessary to maintain robustness in S-phase duration and seems to be of paramount importance, since it also contributes to the maintenance of DNA replication, allowing the survival of this divergent parasite.

## Materials and Methods

### Trypanosomatid culture and growth curves

*T. brucei* procyclic forms (TREU927) were cultured at 28 °C in SDM79 medium supplemented with 10% (v/v) fetal bovine serum. For the growth curves, parasite cultures were initiated with 1. 10^6^ cells.mL^−1^ (daily curves) or with 10 × 10^6^ cells.mL^−1^ (hourly curves), and cells were harvested and counted until they reached the stationary phase. It is worth mentioning that all experimental data presented here were performed using *T. brucei* asynchronous cultures, since agents commonly used to synchronize trypanosomatid cultures (*e.g*., hydroxyurea) could generate genomic stress, consequently introducing a bias in our analysis^78–80^.

### EdU incorporation assays and ‘click’ chemistry reaction

Exponentially growing parasites were incubated with 100 µM 5-ethynyl-2′-deoxyuridine (EdU) (ThermoFisher Scientific) for the time required according to each assay (ranging between 1–2.25 h) at a species-specific temperature (28 °C for *T. brucei*). The parasites were then harvested by centrifugation at 2,500 *g* for 5 min, washed three times in 1x PBS (137 mM NaCl, 2.7 mM KCl, 10 mM Na_2_HPO_4_, and 2 mM KH_2_PO_4_, pH 7.4), and the pellet was resuspended in 200 µL of the same buffer solution. Afterward, 100 µL of the cell suspension was loaded onto poly-L-lysine pretreated microscope slides (Tekdon), fixed for 20 min using 4% sterile paraformaldehyde (Merck) diluted in 1x PBS, washed three times with 1x PBS, and then washed three times with 3% BSA (Sigma-Aldrich) diluted in 1x PBS. Then, parasites were permeabilized for 10 min with 0.1% sterile Triton X-100 (Sigma Aldrich) diluted in 1x PBS, washed three times with 1x PBS, and then washed three times with 3% BSA in 1x PBS. To detect incorporated EdU, we used Click-iT EdU detection solution for 45 min and protected the reaction from light. The Click-iT EdU detection mix solution consisted of 25 µL of 500 mM ascorbic acid (C_6_H_8_O_6_), 5 µL of 100 mM copper sulfate (CuSO_4_), 2.5 µL of Alexa Fluor azide 488 (ThermoFisher Scientific), and 467.5 µL of distilled water (for details about the EdU procedure, see^18^). Finally, the parasites were washed five times with 1x PBS. Vectashield Mounting Medium (Vector) containing 4′,6-diamidino-2-phenylindole dihydrochloride (DAPI) was used as an antifade mounting solution and to stain nuclear and kinetoplast DNA. Images were acquired using an Olympus Bx51 fluorescence microscope (100x oil objective) attached to an EXFO Xcite series 120Q lamp and a digital Olympus XM10 camera with camera controller software Olympus Cell F (Olympus, Japan). Images were further analyzed using ImageJ software (National Institutes of Health) to count the numbers of EdU-positive parasites, and the percentage of proliferating parasites was calculated for each sample relative to the total number of DAPI-positive parasites.

### Methods for analysis of the cell cycle

Formaldehyde-fixed and DAPI-stained exponentially growing procyclic forms of *T. brucei* were examined under an Olympus BX51 fluorescence microscope (Olympus) (100x oil objective) to observe the profile of organelles containing DNA (nucleus and kinetoplast). To estimate the duration of cytokinesis (C), we used the Williams (1971) Equation^19^:

1 $$x=\frac{\mathrm{ln}(1-y/2)}{-\alpha }$$

where x is the cumulative time within the cycle until the end of the stage in question, y is the cumulative proportion of cells up to and including the stage in question (expressed as a fraction of one unit), and α is the specific growth rate.

To estimate the G2 + M phases length, we added EdU in each culture medium containing the parasites for analysis and collected them every 15 min until a parasite containing two EdU-labeled nuclei (2N2K) was observed. Also, to estimate the S-phase duration, we used an EdU pulse (1 h) and measured the proportion of cells EdU-labeled. The duration of S phase was estimated according to the Stanners and Till (1960) Equation^20^:

2 $$S=\frac{1}{\alpha }\,\mathrm{ln}[L+{e}^{\alpha (Z)}]-(Z+t),$$

where *L* is the proportion of cells EdU-labeled, α = ln 2.T^−1^ (*T* = doubling time expressed in hours), Z = G2 + M + cytokinesis, and *t* is the duration of the EdU labeling period in hours.

Finally, the duration of the G1 phase was estimated by the difference between the doubling time and the sum of the remaining phases.

### Estimation of the minimum number of origins required to complete S phase

To determine the minimum number of origins needed to replicate an entire chromosome, we developed an equation. This formula uses the S-phase duration (*S*), the size of the chromosome in question (*N*), and the replication rate (*v*). The lower bound *mo* for the number of origins required to replicate an entire chromosome is given by:

3 $$mo=\lceil \frac{N}{2.v.S}\rceil ,$$

Of note, if the right-hand side of the formula results in a fraction of a unit, then the next higher integer unit must be taken as the result of the formula, which is represented by the ceiling function ($\lceil \rceil$). In Theorem S1 (Supplementary material), we prove the correctness of this equation.

We used up-to-date data available in TriTrypDB database (www.tritrypdb.org) and data reported in recent studies as parameters for the formula^3,17^. We then compared the obtained results with the origins mapped by the MFA-seq technique, presented in another study^16^.

### AT content enrichment analysis and probability landscape for origin firing

AT-content distribution (Fig. S1, red lines) was computed for *T. brucei* TREU927 through an in-house Perl program that split each chromosome into sets of 1,000 bp-sized bins and computed the AT content of each bin.

The probability landscape for origin firing (Fig. S1, blue lines) was generated by assigning to each base pair a probability of origin firing. This probability was derived from a marker frequency analysis coupled with deep sequencing (MFA-seq)^16^, whose processed results were stored in TriTrypDB. For each chromosome, its corresponding MFA-seq data are composed of a thousand equally sized bins. Each bin has a positive real value. Thus, we mapped these real values into a probability value within the interval [0, 1]. In doing so, for each chromosome, we carried out a linear transformation in which we took into account the minimum and maximum bin values, thus yielding our probability landscape.

### Analytical solution for deterministic DNA replication with constitutive origins

Initially, we considered only the origins mapped by the MFA-seq assay^16^, aiming to reconcile those data with the experimentally observed S-phase duration (Fig. 1) and two replication rates measured previously^3,17^. To this end, we derived an analytical solution for the lower-bound required time for DNA replication that relies solely on a set Θ = {θ_1_, …, θ_|Θ|_} of constitutive origin locations, the chromosome size *N* and the replication rate *v*:

4 $$T({\Theta },1,N)\ge ma{x}_{1\le i\le |{\Theta }|+1}\{\frac{1}{2.v}({\theta }_{i}-{\theta }_{i-1})\},{\theta }_{0}=-\,{\theta }_{1,}{\theta }_{|{\Theta }|+1}=2N-{\theta }_{|{\Theta }|}.$$

In Theorem S2 (Supplementary material), we prove the correctness of Eq. 4.

### Simulation of a stochastic dynamic model for replication-transcription conflicts

To carry out simulations of S-phase dynamics in *T. brucei*, we developed a simulator whose main procedures are presented in Fig. S6. For each simulation, we constrained the number of available replisomes during a given simulation (parameter *F*) in a manner similar to the procedure adopted in another meticulous study^27^.

A simulation round starts with all binary vectors (Fig. S2) filled with zeros (*i.e*., all nucleotides are nonreplicated). After that, at the beginning of each iteration, the simulator verifies whether there are replisomes attached to the chromosomes: if there are, then the simulator advances them, filling the section of the binary vector that was replicated. The simulator also evaluates and resolves the replication-transcription conflicts that eventually arise. In the sequence, if the number of activated origins is smaller than or equal to *F -* 2, then one nucleotide of the whole genome is drawn with uniform probability. Origin firing is decided by verifying a given nucleotide probability in the origin firing probability landscape (Fig. S1). In simulations with transcription, in some iterations, RNAP binds onto the beginning of all polycistronic regions, which depends on a parameter called frequency of constitutive transcription. A simulation round ends when the genome is completely replicated or if it reaches a given replication time threshold. The latter might be necessary for replication-transcription conflicts assays since in some simulations the required time for replication completion might be far higher than the mean S-phase duration, which means that the simulated organism is not viable.

A priori information was organized using the SQLite relational database manager (www.sqlite.org). The model simulator was originally implemented in the Python 3 programming language (www.python.org) using the NumPy scientific computing library (www.numpy.org). We also implemented a high-performance version of this simulator, which was coded in C++ programming language. Both source codes are under the GNU GLPv3 license and can be obtained for free at github.com/msreis/ReDyMo (Python version) and github.com/msreis/ReDyMo-CPP (C++ version).

### Nuclear run-on assay to detect transcription throughout the cell cycle

This assay was carried out as described by Hiraiwa *et al*.^81^. The approach to inhibit the transcription is optimized for RNA polymerase II (RNAP II) activity, which mainly transcribes messenger RNA (mRNA). Briefly, the parasites were washed with buffer A (150 mM sucrose, 20 mM L-glutamic acid potassium, 20 mM HEPES-KOH pH 7.7, 3 mM MgCl_2,_ 1 mM DTT) and permeabilized with 250 µg/mL LPC (L-α-lysophosphatidylcholine) in the same buffer. Then, the parasites were resuspended in transcription buffer (150 mM sucrose, 20 mM L-glutamic acid potassium, 20 mM HEPES-KOH pH 7.7, 3 mM MgCl_2,_ 1 mM DTT, 0.6 mg/mL creatine kinase, 25 mM creatine phosphate, 8 U RNase Out, 10 µg/mL leupeptin, 4 mM ATP, 2 mM GTP, 2 mM CTP, 2 mM BrUTP), and *in vivo* transcription was performed at 28 °C for 15 min under gentle agitation. The reaction was stopped by the addition of buffer A, and the parasites were recovered for immunolabeling with anti-bromodeoxyuridine (anti-BrdU antibody) (1:200) and an anti-rat secondary antibody conjugated to Alexa Fluor 488 (1:500). After that, the parasites were resuspended in 1x PBS containing 2 µg.mL^−1^ DAPI and 10 µg.mL^−1^ RNase A for flow cytometry analysis. Data were collected by flow cytometry (FACSCanto II, BD Biosciences, San Jose, CA, USA). The Alexa Fluor 488 fluorochrome (detection of nascent RNA) was excited with a blue laser (488 nm), and the emitted light was collected with a 530/30 bandpass filter. The DAPI stain (DNA content analysis) was excited with a violet laser (405 nm) and collected with a 450/40 bandpass filter. No compensation was needed. The establishment of the cutoff was determined based on the non-labeled group (data not shown). The data were analyzed using FlowJo software (BD, USA) version 10.0.1.

Moreover, the nuclear run-on assay was carried out with parasites previously treated with 75 μg/mL α-amanitin (an irreversible transcription inhibitor) for 4 min to inhibit RNAP II transcription, as described by Hiraiwa *et al*.^81^.

### Immunofluorescence assays (IFA)

Exponentially growing procyclic forms of *T. brucei* were harvested by centrifugation (~5.10^6^ parasites) at 2,500 *g* for 5 min, washed three times with 1x PBS, fixed for 10 min using sterile 4% paraformaldehyde (Merck, Germany) diluted in 1x PBS, and washed again with 1x PBS. Of note, for the IFA involving γH2A and R-loops, the procyclic forms of *T. brucei* were previously treated with α-amanitin and/or treated with ionizing radiation (50 Gy), or treated with exogenous RNase H, as described in previous studies^43,82^. After that, these samples were treated in the same manner as described previously, *i.e*., harvested by centrifugation, washed, fixed, and washed again.

Washed parasites were homogenized in 1x PBS and added for 15 min to Teflon-coated microscope slides (Tekdon). Parasites were then washed three times on slides for two min each with blocking solution (4% BSA in 1x PBS) and permeabilized for 10 min with 0.1% Triton X-100 diluted in 1x PBS. Samples were then incubated at room temperature for 2 h with antisera solution containing anti-γH2A polyclonal rabbit antibodies diluted at 1/1000 in 4% BSA, together with anti-R-loop antibody (DNA:RNA hybrid monoclonal mouse antibody, clone S9.6 – Kerafast) diluted at 1/50 in 4% BSA. After that, parasites were washed three times on slides and incubated for 1 h with a goat anti-rabbit IgG secondary antibody conjugated to Alexa Fluor 555 (Life Technologies) diluted at 1/500 in 1% BSA, together with a goat anti-mouse IgG secondary antibody conjugated to Alexa Fluor 488 (Life Technologies), also diluted at 1/500 in 1% BSA. Afterward, parasites were washed five times, and Vectashield Mounting Medium (Vector) containing 4′,6-diamidino-2-phenylindole dihydrochloride (DAPI) was used as an antifade mounting solution and to stain nuclear and kinetoplast DNA. Simple immunofluorescence images were acquired using an Olympus Bx51 fluorescence microscope (100x oil objective) attached to an EXFO Xcite series 120Q lamp and a digital Olympus XM10 camera with Olympus Cell F camera controller software. The intensity of images captured was estimated using Olympus Cell F tools for 100 cells per sample. When necessary, images were superimposed using Olympus Cell F software. Alternatively, double-immunofluorescence images were analyzed using Olympus IX81 microscope and acquired through a Z-series at a thickness of 0.20 μm (10–12 layers) using a 100X 1.35NA lens, a disk scanning unit (DSU) that provides confocal-like images using a white light, an arc excitation source, and a CCD camera (Olympus). After the acquisition, the images were improved by deconvolution using Autoquant software (Media Cybernetics, Rockville, MD, USA), version X2.1.

### DNA combing

Exponentially growing procyclic forms of *T. brucei* [non-treated (control) and α-amanitin treated] were grown to a concentration of ~1.10^7^ parasites.mL^−1^ and sequentially labeled with two thymidine analogs: 5-iodo-2′-deoxyuridine (IdU, Sigma) at 100 μM for 20 min and 5-chloro-2′-deoxyuridine (CldU, Sigma) at 100 μM for 20 min, without an intermediate wash. After labeling, the cells were immediately harvested by centrifugation at 2,500 *g* for 5 min at 4 °C, washed once with cold 1x PBS and resuspended in 100 μL of 1x PBS with 1% low-melting agarose in order to embed cells in agarose plugs. Then, the plugs were incubated in 500 μL of lysis solution (0.5 M EDTA, pH 8.0, 1% N-lauryl-sarcosyl, and 4 μg.mL^−1^ proteinase K) at 50 °C for 24 h. Next, fresh lysis solution was added, and the plugs were incubated for another 24 h. The plugs were carefully washed several times using 0.5 M EDTA, pH 8.0, to propitiate the complete removal of digested proteins and other degradation products. Protein-free DNA plugs were then stored in 0.5 M EDTA, pH 8.0, at 4 °C or used immediately. Plug samples were washed in TE buffer (10 mM Tris-HCl, pH 8.0; 1 mM EDTA pH 8.0), resuspended in 1 mL of 0.5 M MES buffer (2-(N-morpholino) ethanesulfonic acid, pH 5.5) and melted at 68 °C for 20 min. The solution was maintained at 42 °C for 10 min and treated overnight with 2 U of β-agarose (New England Biolabs). After digestion, 1 mL of 0.5 M MES was added carefully to the DNA solution, and then DNA fibers were regularly stretched (2 kb.μm^−1^) on appropriate coverslips as described previously^83^.

Combed DNA was fixed onto coverslips at 65 °C for at least 2 h, denatured in 1 M NaOH for 20 min and washed several times in 1x PBS. After denaturing, coverslips containing the DNA fibers were blocked with 1% BSA diluted in 1x PBS. Immunodetection was performed using primary antibodies (mouse anti-BrdU antibody clone B44 – 1/20 dilution, Becton Dickinson; and rat anti-BrdU antibody clone BU1/75 (ICR1) – 1/20 dilution, Abcam) diluted in 1% BSA and incubated at 37 °C in a humid chamber for 1 h. Of note, mouse anti-BrdU reacts with IdU and BrdU^84,85^, and the rat anti-BrdU antibody cross-reacts with CldU but does not cross-react with thymidine or IdU^85^. Next, the coverslips were incubated with goat anti-rat Alexa 488 secondary antibodies (1/20 dilution, Life Technologies) and with goat anti-mouse Alexa 568 antibodies (1/20 dilution, Life technologies). Each incubation step with antibodies was followed by extensive washing with 1x PBS. Then, DNA immunodetection was performed using an anti-ssDNA antibody (1/50 dilution, Chemicon) and goat anti-mouse Alexa 350 (1/10 dilution, Life Technologies). Coverslips were then mounted with 20 μl of Prolong Gold Antifade Mountant (ThermoFisher), dried at room temperature for at least 2 h and processed for image acquisition using an Olympus BX51 fluorescence microscope (100x oil objective) attached to an EXFO Xcite series 120Q lamp and a digital Olympus XM10 camera with Olympus camera controller software. When necessary, images were superimposed using the software Olympus Cell F (Olympus, Tokyo, Japan). The observation of longer DNA fibers required the capture of adjacent fields or the use of a fluorescence microscope equipped with a motorized stage that enabled the scanning of slides with high precision. Fibers < 100 kb were excluded from the analysis. The percentage of origins activated during the thymidine pulses was measured manually using ImageJ software among the patterns of DNA fibers (Fig. 7B) collected randomly. Statistical analyses of origin density were performed using Prism 5.0 (GraphPad). Using the DNA fibers that allowed the calculations of the replication speed (n = 42), we were able to estimate the average replication rate in control (non-treated) and α-amanitin-treated groups using the formula $\,(\mathop{\sum }\limits_{1}^{42}lenght/42)/time\,of\,CldU\,pulse$. Three independent combing experiments were performed for the analysis presented in this study.

### S-phase length analysis using the measurement of cytokinesis-labeled nuclei

To estimate the duration of S phase, we used an EdU pulse (100 μM during 15 min) in an exponentially growing culture of *T. brucei* (control and α-amanitin-treated). The parasites were then harvested every 15 min, washed three times in 1x PBS, and fixed for 10 min with 3.7% sterile paraformaldehyde (Merck) diluted in 1x PBS. This same approach was also performed in the presence of α-amanitin, *i.e*., in the absence of transcription. Afterward, the parasites were washed, added to a slide (Tekdon) containing 0.1% poly-L-lysine, permeabilized for 10 min with 0.1% sterile Triton X-100 (Sigma) diluted in 1x PBS, and treated using ‘click’ chemistry to detect EdU (for more details, see EdU incorporation assays and ‘click’ chemistry reaction section described previously). After the addition of Vectashield Mounting Medium containing DAPI (Vector) to stain nuclei (N) and kinetoplasts (K), we sealed the slides, and we measured the percentage of parasites containing two labeled nuclei (cytokinesis). The duration of S phase starts when the first cytokinesis-labeled cell appears, extends throughout the period of detection for all cytokinesis-labeled cells and ends when some unlabeled cells appear again. Of note, this assay was carried out according to a protocol described in a previous study^15^.

### Statistical analysis

Assays for each analysis presented herein were performed at least in duplicate in three independent experimental and biological conditions, and the data were analyzed using Prism 5 software (GraphPad). Quantitative data are expressed as the mean ± standard error, and the results were statistically analyzed using the Student’s t-test. Differences with *p* values < 0.05 were considered statistically significant.

## Supplementary information

Supplementary Dataset

## Data Availability

A priori information of our *in silico* analyses was organized using the SQLite relational database manager (www.sqlite.org). The model simulator was originally implemented in the Python 3 programming language (www.python.org) using the NumPy scientific computing library (www.numpy.org). Also, we implemented a high-performance version of our simulator coded in C++ programming language. Both source codes are under the GNU GLPv3 license and can be obtained for free at github.com/msreis/ReDyMo (Python version) and github.com/msreis/ReDyMo-CPP (C++ version).
