# Supplementary material for: Transcription activity contributes to the firing of non-constitutive origins in African trypanosomes helping to maintain robustness in S-phase duration
Source: Sci Rep. 2019 Dec 6;9:18512. doi: 10.1038/s41598-019-54366-w (PMC6898680; doi:10.1038/s41598-019-54366-w)
Supplement: Supplementary file 1 — Supplementary Dataset [file 41598_2019_54366_MOESM1_ESM.pdf]

Supplementary Material of

Transcription activity contributes to the firing of non-constitutive origins in African trypanosomes helping to maintain robustness in S-phase duration

Marcelo S. da Silva, Gustavo R. Cayres-Silva, Marcela O. Vitarelli, Paula A. Marin, Priscila M. Hiraiwa, Christiane B. Araújo, Bruno B. Scholl, Andrea R. Ávila, Richard McCulloch, Marcelo S. Reis, and Maria Carolina Elias

Contents

1 Correctness of Equations 3 and 4 **2**

1.1 Minimum number of origins required to complete S phase 2

1.2 Lower-bound time for DNA replication with constitutive origins only 3

2 Supplementary Figures **5**

3 Supplementary Tables **11**

1 Correctness of Equations 3 and 4

In this section, we present proofs of correctness for Equations 3 and 4 of the main paper. For both of these equations, we assume that the replication fork speed v has a negligible variance along the S-phase duration.

1.1 Minimum number of origins required to complete S phase

The lower-bound number of origins to replicate an entire chromosome, given as a function of chromosome size, S-phase duration and replication fork speed was presented both in Table 1 and Figure 2 of the main paper. The following theorem guarantees the correctness of that equation.

Theorem S1. *Let v be the replication fork average speed, S be the measured S-phase duration, and N be the chromosome size. The lower bound for the number of origins required to replicate the entire chromosome is given by:*

$$oe \geq \left\lceil \frac{N}{2\ v\ S} \right\rceil . \tag{3}$$

Proof. Consider two cases for Equation 3:

- $N \leq 2vS$: in this case, we set a single origin in the middle of the chromosome. Since each fork must replicate $\frac{N}{2}$ bases with velocity v and in at most S time, we have:

$$oe = \underbrace{1 \geq \left\lceil \frac{\frac{N}{2}}{vS} \right\rceil}_{\text{Since } N \leq 2vS} = \left\lceil \frac{N}{2vS} \right\rceil .$$

- $N > 2vS$: In this case, we assume for this chromosome a minimal set Θ of replication origins such that $|\Theta| \geq 2$. We also assume that the chromosome is divided into $|\Theta| + 1$ pieces, and that each piece demands exactly S time to replication. These assumptions imply that the size of the first and the last pieces is $\frac{N}{2|\Theta|}$ each, while the size of the remaining $|\Theta| - 1$ pieces is $\frac{N}{|\Theta|}$ each (if we slide any origin either to right or to the left, then the piece of the opposite side will demand a time greater than S, a contradiction on the given S-phase duration). Now, let us apply Equation 4, which returns a lower bound

time as a function of a set of origins, chromosome size and fork velocity:

$$\begin{aligned} T(\Theta, \langle 1, N \rangle) &\geq \max_{1 \leq i \leq |\Theta|+1} \left\{ \frac{1}{2v} (\theta_i - \theta_{i-1}) \right\} \\ &= \frac{1}{2v} \left(\theta_2 - \theta_1 \right) && \text{(Equivalent to a remaining piece)} \\ &= \frac{1}{2v} \left(\frac{N}{|\Theta|} \right) && \text{(The size of a remaining piece)} \\ &= \frac{N}{2v} \left(\frac{1}{|\Theta|} \right). \end{aligned}$$

Observe that the last right-hand side of the equations above is equal to S, since each piece requires the same amount of time to replicate. Thus, we have:

$$\begin{aligned} S &= \frac{N}{2v} \left(\frac{1}{|\Theta|} \right) \\ |\Theta| \ S &= \frac{N}{2v} \left(\frac{1}{\cancel{|\Theta|}} \right) \cancel{|\Theta|} \\ |\Theta| \ S &= \frac{N}{2v} \\ \frac{1}{\cancel{S}} \ |\Theta| \ \cancel{S} &= \frac{N}{2v} \ \frac{1}{S} \\ |\Theta| &= \frac{N}{2v \ S}. \end{aligned}$$

Finally, once $|\Theta|$ is an integer greater or equal to 2, it holds that:

$$oe \geq |\Theta| = \lceil |\Theta| \rceil = \left\lceil \frac{N}{2v \ S} \right\rceil.$$

□

1.2 Lower-bound time for DNA replication with constitutive origins only

We start with formal definitions of chromosome and also of constitutive origins. A *chromosome* $\langle 1, N \rangle$ is an ordered set of the first N positive integers. A *constitutive origin* θ is a positive integer in $\langle 1, N \rangle$. The correctness of Equation 4 is assured by the following theorem.

Theorem S2. *Let v be the replication fork average speed. For a given chromosome of size N, let $\Theta = \{\theta_1, \ldots, \theta_{|\Theta|}\}$ be a set of constitutive origins, $\theta_1 < \ldots < \theta_{|\Theta|}$. The lower-bound time for complete chromosome replication with origins in Θ only is:*

$$T(\Theta, \langle 1, N \rangle) \geq \max_{1 \leq i \leq |\Theta|+1} \left\{ \frac{1}{2v} (\theta_i - \theta_{i-1}) \right\}, \tag{4}$$

where $\theta_0 = -\theta_1$ and $\theta_{|\Theta|+1} = 2N - \theta_{|\Theta|}$.

Proof. This proof is an induction on $|\Theta|$. If $|\Theta| = 1$, then the firing of the single origin yields

two forks. One of them slides, at velocity v, from location θ_1 to 1; the other one slides, also at velocity v, from θ_1 to N. Thus, we have:

$$\begin{aligned}
T(\Theta, \langle 1, N\rangle) &\geq T(\{\theta_1\}, \langle 1, N\rangle) \\
&\geq \max\left\{\frac{\theta_1}{v}, \frac{N-\theta_1}{v}\right\} \\
&\geq \max\left\{\frac{2\theta_1}{2v}, \frac{2(N-\theta_1)}{2v}\right\} \\
&\geq \max\left\{\frac{\theta_1+\theta_1}{2v}, \frac{2N-2\theta_1}{2v}\right\} \\
&\geq \max\left\{\frac{\theta_1-(-\theta_1)}{2v}, \frac{(2N-\theta_1)-\theta_1}{2v}\right\} \\
&\geq \max\left\{\frac{\theta_1-\theta_0}{2v}, \frac{\theta_{|\Theta|+1}-\theta_{|\Theta|}}{2v}\right\} \\
&\geq \max_{1\leq i\leq |\Theta|+1}\left\{\frac{1}{2v}(\theta_i-\theta_{i-1})\right\}.
\end{aligned}$$

Now, consider $|\Theta| > 1$. Let us set $\Omega = \{\theta_{|\Theta|}\}$ and $A = \Theta - \Omega$. Moreover, we define $N_A = \frac{\theta_{|\Theta|} - \theta_{|\Theta|-1}}{2}$ and $N_\Omega = N - N_A$. This is equivalent to split the original instance into two pieces: one contains the last origin ($\theta_{|\Theta|}$) and the final stretch of the chromosome, which goes from halfway between $\theta_{|\Theta|} - 1$ and $\theta_{|\Theta|}$ (that is, where the encounter of replisomes from these two origins takes place) and N; ant the second one with both the remaining origins ($\theta_1, \ldots, \theta_{|\Theta|-1}$) and the first stretch of the chromosome. Thus, we have:

$$\begin{aligned}
T(\Theta, \langle 1, N\rangle) &\geq T(\{\theta_1, \ldots, \theta_{|\Theta|}\}, \langle 1, N\rangle) \\
&\geq \max\Big\{T(A, \langle 1, N_A\rangle), T(\Omega, \langle 1, N_\Omega\rangle)\Big\} \\
&\geq \max\left\{\max\left\{\frac{\alpha_1}{v}, \ldots, \frac{N_A-\alpha_{|A|}}{v}\right\}, \max\left\{\frac{\omega_1}{v}, \frac{N_\Omega-\omega_{|\Omega|}}{v}\right\}\right\} \\
&\geq \max\left\{\underbrace{\max_{1\leq i\leq |A|+1}\left\{\frac{1}{2v}(\alpha_i-\alpha_{i-1})\right\}}_{\text{induction hypothesis}}, \underbrace{\max_{1\leq i\leq |\Omega|+1}\left\{\frac{1}{2v}(\omega_i-\omega_{i-1})\right\}}_{\text{as in the case of } |\Theta|=1}\right\} \\
&\geq \max\left\{\max_{1\leq i\leq (|\Theta|-1)+1}\left\{\frac{1}{2v}(\theta_i-\theta_{i-1})\right\}, \max_{1\leq i\leq (1)+1}\left\{\frac{1}{2v}(\theta_{|\Theta|+i-1}-\theta_{|\Theta|+i-2})\right\}\right\} \\
&\geq \max\left\{\max_{1\leq i\leq |\Theta|}\left\{\frac{1}{2v}(\theta_i-\theta_{i-1})\right\}, \max_{|\Theta|\leq i\leq |\Theta|+1}\left\{\frac{1}{2v}(\theta_i-\theta_{i-1})\right\}\right\} \\
&\geq \max\left\{\max_{1\leq i\leq |\Theta|+1}\left\{\frac{1}{2v}(\theta_i-\theta_{i-1})\right\}\right\} \\
&\geq \max_{1\leq i\leq |\Theta|+1}\left\{\frac{1}{2v}(\theta_i-\theta_{i-1})\right\}.
\end{aligned}$$

□

2 Supplementary Figures

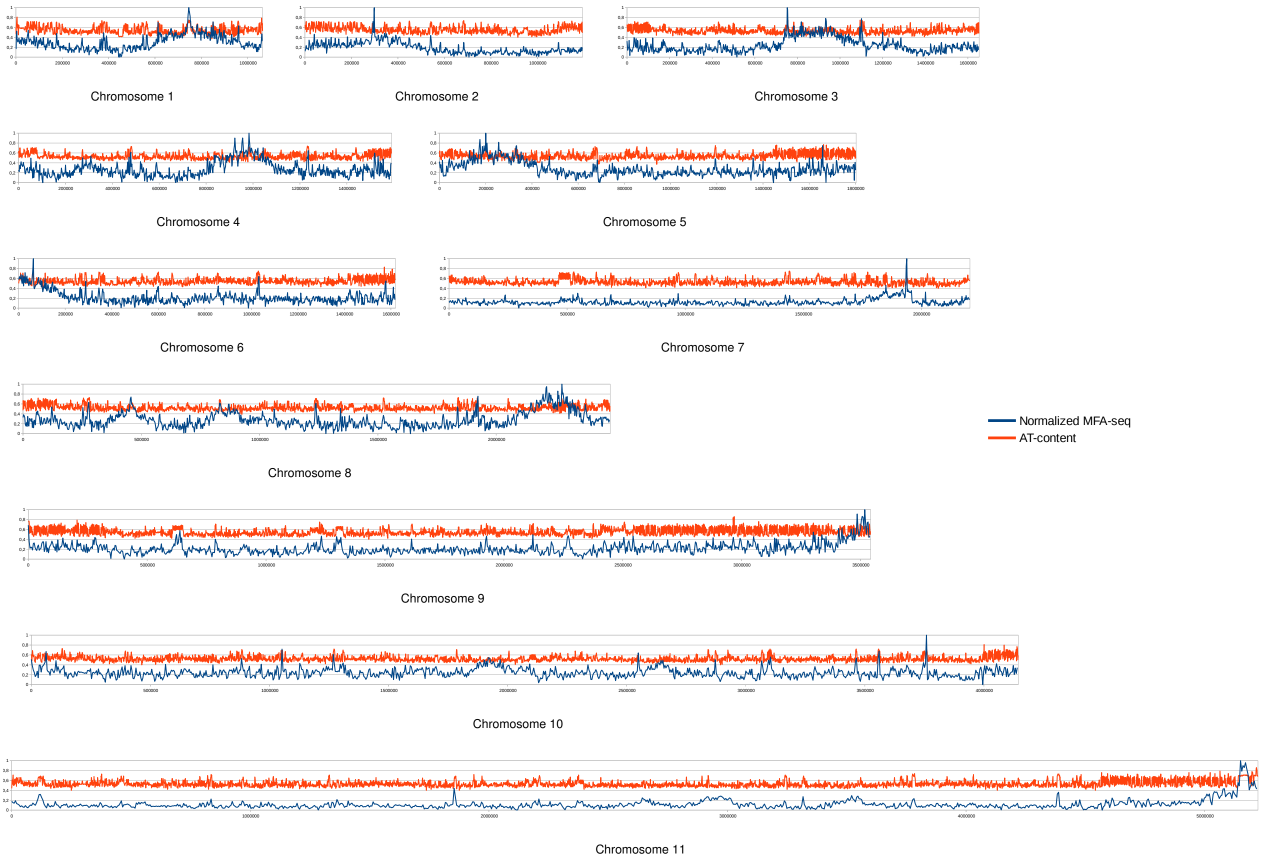

Figure S1. Probability landscapes for origin firing of the core-genome of *T. brucei* TREU927. For each chromosome, MFA-seq data downloaded from TriTrypDB database were normalized through a linear transformation (blue lines). The obtained patterns are then compared with AT-content distribution of their respective chromosomes (red lines).

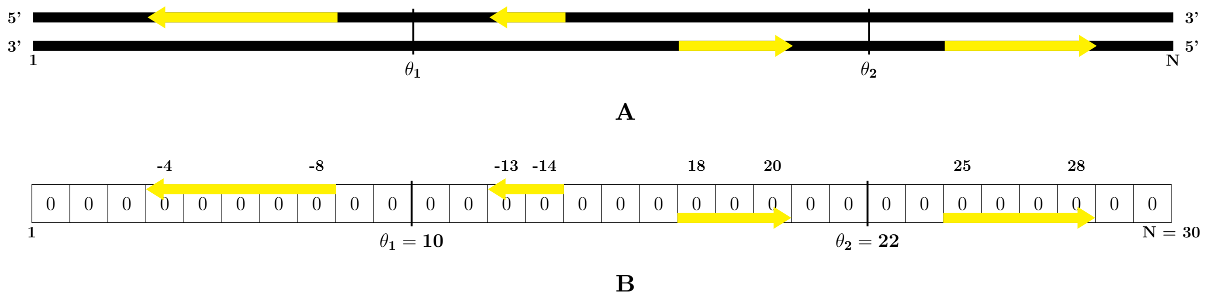

Figure S2. Chromosome representation in our dynamic model. A. Example of chromosome description, which includes its size N, locations of constitutive origins (in this example we have two of them, θ_1 and θ_2) and location of polycistronic regions in both strands (yellow arrows). **B.** Example of chromosome description mapping into a binary vector, where "0" and "1" means that a given nucleotide is non-replicated and replicated, respectively. In this example, we assume $N = 30$ nucleotides. Additional data are stored to give coordinates of constitutive origins and polycistronic regions; in the case of this latter, negative values mean that it is located in the complementary strand in respect to the initial nucleotide.

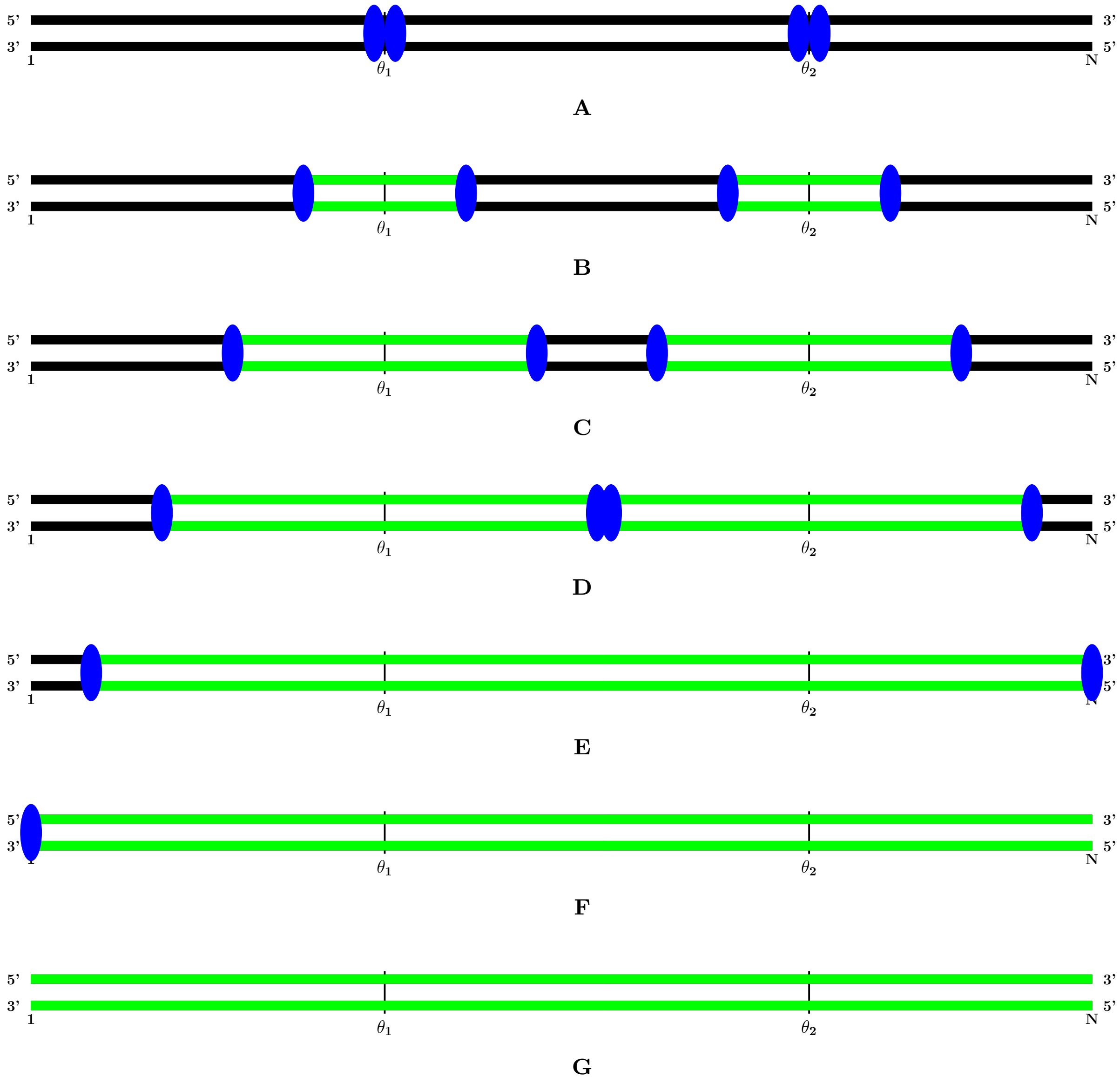

Figure S3. Example of DNA replication dynamics. A. Two origins (θ_1 and θ_2) are fired, with two replisomes (blue ellipses) binding on each origin. **B–C.** For each fired origin, its respective pair of replisomes slide on the opposite direction to each other, leaving behind replicated chromosome (in green). **D–E.** If there is a head-to-head collision between two replisomes, they unbind the chromosome. **F–G.** The replisome also unbinds the chromosome if it reaches an extremity of the chromosome.

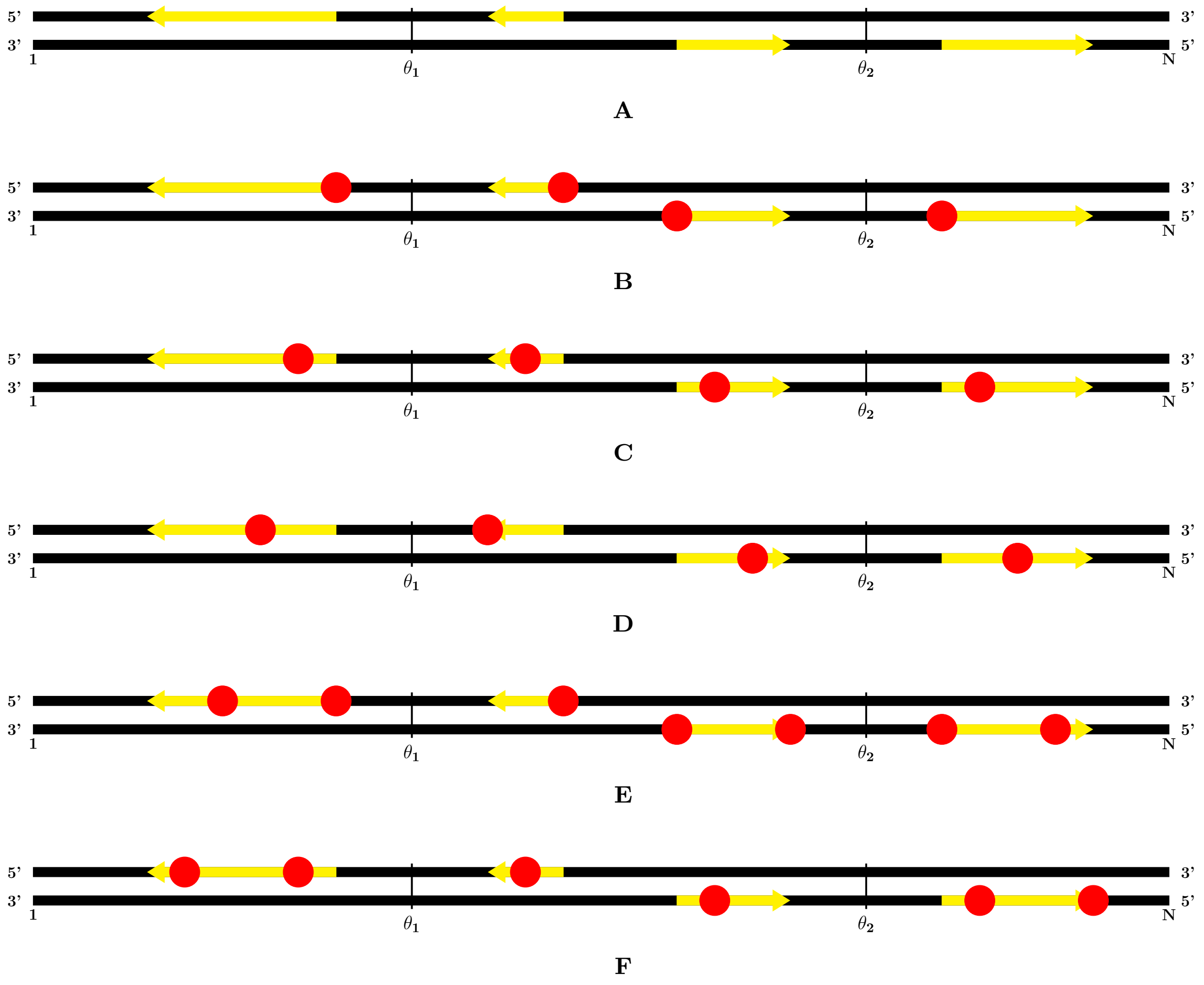

Figure S4. Example of DNA transcription dynamics. A–B. At a given constant frequency, a RNA polymerase (RNAP) binds to the beginning of each polycistronic region. At each iteration, all RNAPs slides on the chromosome at a constant velocity (**B–F**). When a RNAP reaches the end of polycistronic region, it releases the chromosome (e.g., compare **E** with **F**, looking at the second rightmost yellow arrow).

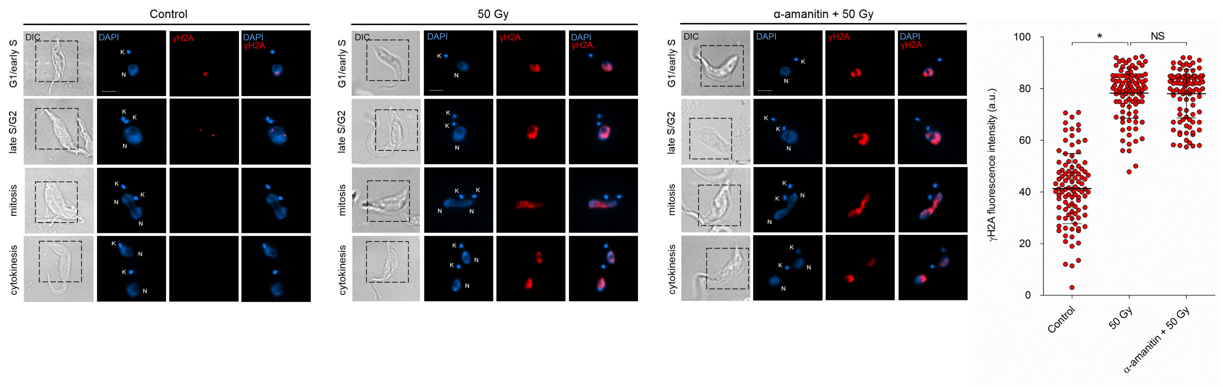

Figure S5. The immediate phosphorylation of H2A does not depend directly on nascent RNAs inhibited by α-amanitin. Distribution profile of γH2A fluoresence throughout the cell cycle of non-treated (control), ionizing radiation (50 Gy) treated, and treated with α-amanitin + 50 Gy parasites. The scale bar on the fluorescence images corresponds to 2 μm. K and N mean kinetoplast and nucleus, respectively. The graph shows the γH2A fluorescence intensity (red) per cell in the samples previously described. Errors bars indicate SD. * and NS mean, respectively, $p < 0.001$ and non-significant using Student's t-test ($n = 100$ cells per group).

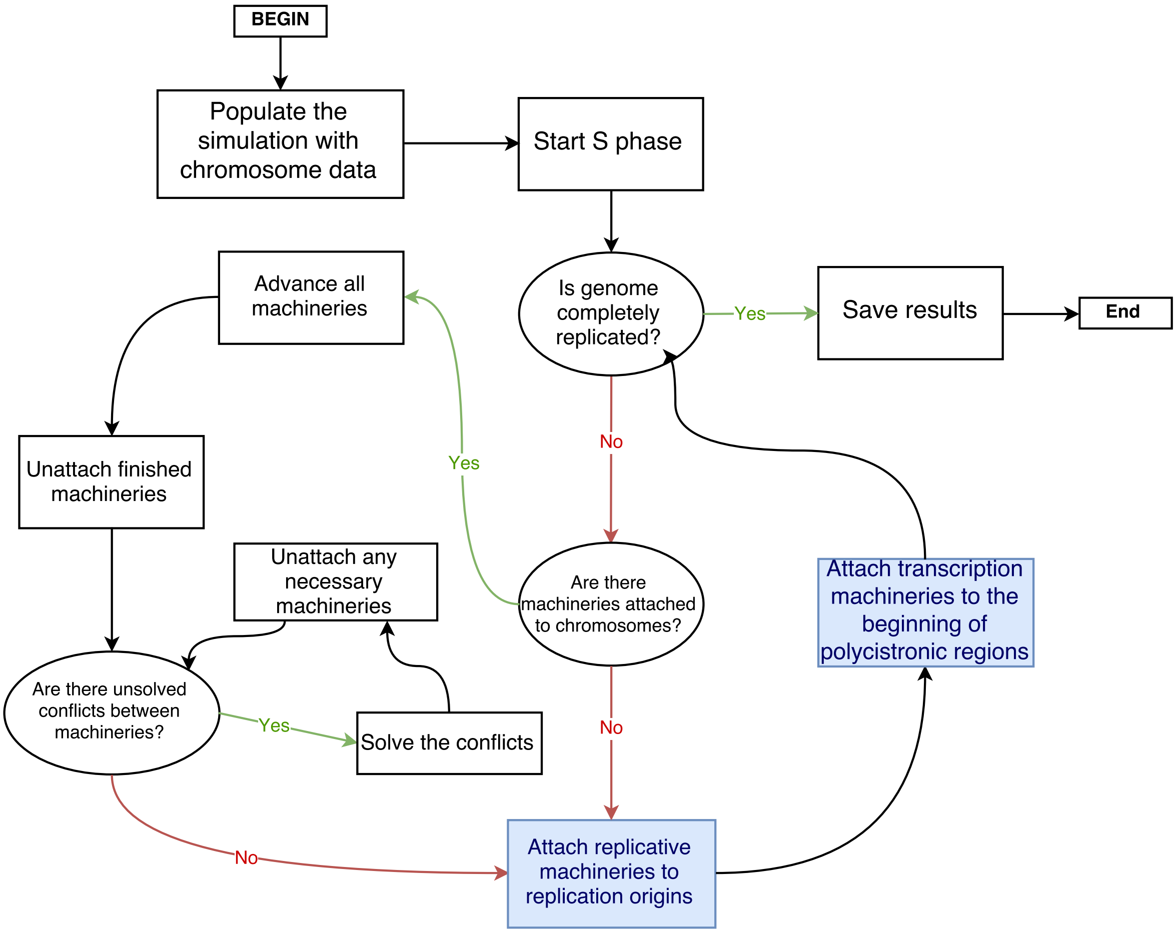

Figure S6. Fluxogram of the DNA replication simulator. In this chart, it is depicted the major steps during a simulation of DNA replication dynamics in *T. brucei* TREU927. The simulator implementation, called ReDyMo, was coded in Python programming language and is available at GitHub: github.com/msreis/ReDyMo. A C++ port of this program is also available at that repository: github.com/msreis/ReDyMo-CPP.

3 Supplementary Tables

Table S1. Lower-bound time for DNA replication in *T. brucei* TREU927 megabase-sized chromosomes, using only putative constitutive origins presented in Tiengwe et al. (2012). These results were obtained using the Equation 4 of the current study. In the case where artificial origins were included in subtelomeric regions, for a chromosome of size N, a pair of origins were included into positions 50 kb and ($N - 50$ kb).

Chromosome number	Replisome velocity (b/sec)	Including putative origin in subtelomeric regions?	Time (sec)
1	61.66	Yes	5163.36
2	61.66	Yes	5080.33
3	61.66	Yes	6726.55
4	61.66	Yes	5080.33
5	61.66	Yes	5226.88
6	61.66	Yes	9950.6
7	61.66	Yes	14068.6
8	61.66	Yes	3566
9	61.66	Yes	10200.66
10	61.66	Yes	11805.23
11	61.66	Yes	13861.97
1	30.66	Yes	10383.99
2	30.66	Yes	10216.99
3	30.66	Yes	13527.69
4	30.66	Yes	10216.99
5	30.66	Yes	10511.73
6	30.66	Yes	20011.55
7	30.66	Yes	28293.22
8	30.66	Yes	7171.54
9	30.66	Yes	20514.45
10	30.66	Yes	23741.37
11	30.66	Yes	27877.66
1	61.66	No	11137.63
2	61.66	No	5080.33
3	61.66	No	14264
4	61.66	No	5178.02
5	61.66	No	7638.31
6	61.66	No	10160.66
7	61.66	No	14068.6
8	61.66	No	7620.48
9	61.66	No	21212.23
10	61.66	No	24421.36
11	61.66	No	28534.84
1	30.66	No	22398.76
2	30.66	No	10216.99
3	30.66	No	28686.17
4	30.66	No	10413.47
5	30.66	No	15361.32
6	30.66	No	20433.99
7	30.66	No	28293.22
8	30.66	No	15325.47
9	30.66	No	42659.69
10	30.66	No	49113.54
11	30.66	No	57386.11

Table S2. Results with the DNA replication model. For each number of available replisomes during simulation (F) and for each transcription period, it was carried out 30 simulations, whose results were averaged. To evaluate S-phase duration robustness in increasing levels of transcription, for each F value, the assay with no transcription was assumed as taking exactly the measured S-phase duration (2.31 hours); thus, the S-phase duration of the remaining assays of this F value were obtained through a normalization using the mean number of iterations. A similar procedure was used to yield the transcription frequency of each assay. AU = arbitrary unit; IOD = inter-origin distance. We highlight in yellow the S-phase duration that is within the interval $[2.07, 2.55]$ (10% above or below the measured S-phase duration). We also highlight in green a replisome velocity (v) that is within either the interval $[55.49, 67.82]$ or the interval $[27.59, 33.72]$ (i.e., 10% above or below the two different measured values for v).

F	Transcription period (# Iterations)	Mean # Iterations (AU)	Dev # Iterations (AU)	Mean IOD (b)	Dev IOD (b)	Median distance between origin and fork stall (b)	Mean distance between origin and fork stall (b)	Dev distance between origin and fork stall (b)	S-phase duration (hours)	Transcription frequency (mHz)	v (b/sec), assuming 1 replicated base per iteration and S-phase duration = 2.31 h
10	∞	2800095.1	30888.4	507657.7	53802.0				2.3	0.0	336.7
10	100000	2817172.0	17064.3	213484.2	18205.9	45233.0	129795.9	187904.0	2.3	3.4	338.8
10	10000	2860804.2	58556.9	141292.4	11247.3	4620.0	72553.5	140539.4	2.4	33.7	344.0
10	1000	3260555.7	1497059.0	121283.6	10205.9	464.0	61239.6	127105.8	2.7	336.7	392.1
10	100	6434736.4	7115102.6	110937.4	9661.3	47.0	57567.4	125154.0	5.3	3367.1	773.8
15	∞	1839820.9	12613.1	368282.7	34977.4				2.3	0.0	221.2
15	100000	1843163.4	7578.8	182898.3	13283.7	40746.0	108899.1	155570.0	2.3	2.2	221.6
15	10000	1866277.9	37641.8	125713.7	10171.4	4356.0	59993.8	118054.2	2.3	22.1	224.4
15	1000	2189960.7	1565243.4	108444.9	8290.3	438.0	50760.7	109193.9	2.7	221.2	263.3
15	100	4740660.0	5990392.1	98182.3	7598.6	44.0	46032.9	101346.1	6.0	2212.4	570.1
20	∞	1366731.4	7473.2	286127.3	23474.3				2.3	0.0	164.3
20	100000	1372061.9	17356.8	159169.6	11235.3	37424.0	90306.8	126946.1	2.3	1.6	165.0
20	10000	1389404.7	42302.8	111555.1	7676.9	4136.0	50544.6	101466.4	2.3	16.4	167.1
20	1000	1641386.5	1274142.6	97189.2	6978.8	418.0	41770.5	91026.6	2.8	164.3	197.4
20	100	3205539.8	4044632.7	89712.3	7281.5	42.0	39025.0	88064.3	5.4	1643.5	385.5
25	∞	1090247.8	4573.0	239136.1	17685.7				2.3	0.0	131.1
25	100000	1092353.0	11973.9	142005.4	9915.8	35337.0	80573.2	112239.2	2.3	1.3	131.4
25	10000	1107750.5	61041.8	101390.8	7316.2	3967.0	42407.8	85779.7	2.3	13.1	133.2
25	1000	1228004.8	358337.7	88298.6	6069.0	401.0	35271.1	79070.5	2.6	131.1	147.7
25	100	3332021.0	5142399.5	82623.7	5673.9	41.0	33935.2	77664.4	7.1	1311.0	400.7
30	∞	905337.8	3478.9	205601.7	17169.5				2.3	0.0	108.9
30	100000	913140.2	76405.2	129295.6	7831.6	33995.5	72276.9	99672.0	2.3	1.1	109.8
30	10000	921835.3	107740.3	92996.2	5906.1	3799.0	36945.2	75958.3	2.4	10.9	110.9
30	1000	1046636.5	730012.3	82149.5	5678.5	390.0	31163.0	70923.4	2.7	108.9	125.9
30	100	2600757.7	3182643.0	76181.5	5010.4	40.0	29446.1	68710.2	6.6	1088.7	312.7
35	∞	775349.0	2635.3	178763.3	12876.4				2.3	0.0	93.2
35	100000	776810.4	4215.0	116340.1	6907.0	30750.0	62210.4	85360.9	2.3	0.9	93.4
35	10000	783515.9	26817.3	86024.9	5522.3	3709.0	33027.3	69313.3	2.3	9.3	94.2
35	1000	901612.9	342192.1	75969.4	4830.5	379.0	27320.6	63657.3	2.7	93.2	108.4
35	100	2072921.1	2912049.2	70961.9	4938.3	39.0	25989.1	61507.7	6.2	932.4	249.3
40	∞	677625.5	2137.2	157214.2	9961.0				2.3	0.0	81.5
40	100000	678935.1	3014.4	106850.2	6210.1	29260.0	57192.6	76962.6	2.3	0.8	81.6
40	10000	686226.7	25534.2	79396.1	4780.5	3558.0	28807.8	60704.9	2.3	8.1	82.5
40	1000	811819.7	375015.4	70829.3	4164.1	372.0	24008.1	57015.7	2.8	81.5	97.6
40	100	2118548.1	4059633.2	66048.7	4377.3	38.0	22850.7	55247.6	7.2	814.8	254.8
45	∞	602177.4	1837.9	142335.6	8838.5				2.3	0.0	72.4
45	100000	603072.6	3099.8	100210.0	5311.3	28598.5	53302.7	69710.4	2.3	0.7	72.5
45	10000	610230.4	30743.2	74854.0	4325.1	3498.0	25849.5	55566.0	2.3	7.2	73.4
45	1000	708700.2	383742.3	66178.0	3954.6	362.0	21093.7	51515.5	2.7	72.4	85.2
45	100	1774159.7	2592117.8	62080.9	3786.4	37.0	20228.0	50085.0	6.8	724.1	213.3
50	∞	541492.5	1574.1	129126.6	7984.0				2.3	0.0	65.1
50	100000	543291.4	6738.4	92334.5	4781.4	26165.0	47789.3	62848.3	2.3	0.7	65.3
50	10000	550079.7	45092.1	70253.9	3948.1	3408.0	23551.7	50220.6	2.3	6.5	66.1
50	1000	628461.0	230268.9	62115.1	3519.6	355.0	18942.1	46911.2	2.7	65.1	75.6
50	100	1591908.7	2144777.9	58432.2	3310.1	36.0	18320.1	46296.7	6.8	651.1	191.4

(Continuation of Table S2.)

55	∞	492248.0	1357.9	118074.5	7226.6				2.3	0.0	59.2
55	100000	493378.3	4812.7	86915.3	4676.7	26484.0	45990.3	58052.1	2.3	0.6	59.3
55	10000	506864.4	158713.5	66324.9	4160.5	3330.0	21430.5	46213.5	2.4	5.9	61.0
55	1000	602750.6	522172.0	58600.7	3401.2	348.0	16803.2	42456.6	2.8	59.2	72.5
55	100	1517570.9	2608599.9	55600.7	2934.0	36.0	16410.2	42150.7	7.1	591.9	182.5
60	∞	451209.0	1287.7	109379.1	5943.1				2.3	0.0	54.3
60	100000	452063.1	1459.0	81340.2	4088.5	24341.0	41737.5	53059.9	2.3	0.5	54.4
60	10000	459041.8	40672.7	62862.0	3256.0	3261.0	19611.7	42967.4	2.4	5.4	55.2
60	1000	562847.8	396259.4	55971.7	3184.0	341.0	15700.9	40274.2	2.9	54.3	67.7
60	100	1196486.0	1557738.1	52805.7	2798.9	35.0	15176.6	39506.2	6.1	542.6	143.9
65	∞	416690.5	1059.0	102317.2	5184.4				2.3	0.0	50.1
65	100000	419022.9	22222.8	76655.0	3848.6	23868.0	39876.6	49548.8	2.3	0.5	50.4
65	10000	419777.9	9012.9	59448.8	3141.7	3212.0	18275.4	39784.3	2.3	5.0	50.5
65	1000	525695.8	369050.6	53309.7	2600.0	338.0	14306.9	37171.1	2.9	50.1	63.2
65	100	1018359.3	1160598.6	50208.6	2870.1	35.0	13627.4	36149.3	5.6	501.1	122.5
70	∞	386820.1	1095.7	95758.1	4472.7				2.3	0.0	46.5
70	100000	387294.1	1624.5	73345.5	3417.7	23965.0	38336.1	46188.7	2.3	0.5	46.6
70	10000	390106.8	5895.2	56481.7	2770.3	3169.0	16760.1	36816.9	2.3	4.7	46.9
70	1000	427303.8	89961.1	50720.7	2775.3	333.0	12945.2	34516.0	2.6	46.5	51.4
70	100	1206053.1	1552662.8	47965.1	2479.4	34.0	12667.1	34083.8	7.2	465.2	145.0
75	∞	361131.6	973.9	89976.4	4888.0				2.3	0.0	43.4
75	100000	361833.5	1803.3	69329.7	3171.4	22021.0	35182.0	42541.5	2.3	0.4	43.5
75	10000	367518.3	25946.0	54162.5	2615.8	3109.0	15684.6	34894.5	2.4	4.3	44.2
75	1000	441436.6	383113.9	48477.1	2373.4	329.0	12118.0	32506.3	2.8	43.4	53.1
75	100	1045756.9	1379431.5	45859.1	2277.6	34.0	11751.4	32170.5	6.7	434.3	125.8
80	∞	338524.0	924.4	84812.5	4027.7				2.3	0.0	40.7
80	100000	339262.9	1117.0	65948.4	2975.9	21418.5	33986.3	40504.9	2.3	0.4	40.8
80	10000	344841.5	35100.9	51539.6	2326.0	3070.0	14686.0	32735.9	2.4	4.1	41.5
80	1000	400331.0	175977.9	46513.5	2154.9	326.0	11130.4	30325.1	2.7	40.7	48.1
80	100	1105893.4	2205852.8	44178.2	2141.4	34.0	10723.7	29898.6	7.5	407.1	133.0
85	∞	318767.5	867.4	80274.4	3999.7				2.3	0.0	38.3
85	100000	319419.2	1202.3	63237.8	2696.7	21246.0	32516.3	37670.2	2.3	0.4	38.4
85	10000	325643.2	38887.5	49549.9	2347.1	3012.0	13561.6	30105.8	2.4	3.8	39.2
85	1000	379827.1	242833.6	44792.9	2059.7	322.0	10426.0	28925.9	2.8	38.3	45.7
85	100	972180.0	1934585.7	42365.1	2177.0	33.0	10032.5	28322.3	7.0	383.3	116.9
90	∞	301024.7	795.0	76673.3	4033.8				2.3	0.0	36.2
90	100000	301498.9	1797.8	61112.4	2944.7	21181.5	31987.4	35991.0	2.3	0.4	36.3
90	10000	309192.1	86882.2	47727.9	2133.8	2996.0	12966.7	29054.6	2.4	3.6	37.2
90	1000	380087.2	330827.9	42883.8	2032.9	320.0	9478.2	26584.1	2.9	36.2	45.7
90	100	941594.0	1290239.6	40785.9	1895.1	33.0	9254.2	26647.2	7.2	362.0	113.2
95	∞	285325.5	807.1	72379.1	3308.8				2.3	0.0	34.3
95	100000	285815.4	911.2	58132.2	2528.1	20459.0	30541.2	34299.6	2.3	0.3	34.4
95	10000	290402.8	29322.6	46101.1	2061.3	2965.0	12387.1	27529.5	2.4	3.4	34.9
95	1000	333917.2	143953.0	41443.8	1924.9	315.0	9081.5	25781.7	2.7	34.3	40.2
95	100	886119.5	1384115.0	39610.9	1855.4	33.0	8808.1	25632.4	7.2	343.1	106.6
100	∞	271169.7	838.2	69229.1	3265.1				2.3	0.0	32.6
100	100000	271578.4	855.7	55786.0	2131.6	19784.5	29263.5	32634.9	2.3	0.3	32.7
100	10000	275506.8	36723.0	44097.7	1839.6	2921.0	11586.8	25738.7	2.3	3.3	33.1
100	1000	319224.5	130987.8	40073.0	1737.7	314.0	8403.7	24306.4	2.7	32.6	38.4
100	100	812367.2	1046166.6	38202.2	1696.7	33.0	8281.0	24266.9	6.9	326.1	97.7
